# High expression of oxidative phosphorylation genes predicts improved survival in squamous cell carcinomas of the head and neck and lung

**DOI:** 10.1038/s41598-020-63448-z

**Published:** 2020-04-14

**Authors:** Mitchell Frederick, Heath D. Skinner, Sawad A. Kazi, Andrew G. Sikora, Vlad C. Sandulache

**Affiliations:** 1 Bobby R. Alford Department of Otolaryngology Head and Neck Surgery, Baylor College of Medicine, Houston, TX United States; 2 Department of Radiation Oncology, UPMC Hillman Cancer Center, Pittsburgh, PA United States; 3 School of Natural Sciences, University of Texas, Austin, TX United States

**Keywords:** Cancer metabolism, Head and neck cancer

## Abstract

Mitochondrial activity is a critical component of tumor metabolism, with profound implications for tumorigenesis and treatment response. We analyzed clinical, genomic and expression data from patients with oral cavity squamous cell carcinoma (OCSCC) in order to map metabologenomic events which may correlate with clinical outcomes and identified nuclear genes involved in oxidative phosphorylation and glycolysis (OXPHOG) as a critical predictor of patient survival. This correlation was validated in a secondary unrelated set of lung squamous cell carcinoma (LUSC) and was shown to be driven largely by over-expression of nuclear encoded components of the mitochondrial electron transport chain (ETC) coordinated with an increase in tumor mitochondrial DNA copy number and a strong threshold effect on patient survival. OCSCC and LUSC patients with a favorable OXPHOG signature demonstrated a dramatic (>2fold) improvement in survival compared to their counterparts. Differential OXPHOG expression correlated with varying tumor immune infiltrates suggesting that the interaction between tumor metabolic activity and tumor associated immunocytes may be a critical driver of improved clinical outcomes in this patient subset. These data provide strong support for studies aimed at mechanistically characterizing the interaction between tumor mitochondrial activity and the tumor immune microenvironment.

## Introduction

The role of mitochondria in tumorigenesis and cancer treatment response remains enigmatic. The initial description of the Warburg effect lead to a parallel conclusion that mitochondria played a minor role in the maintenance and survival of tumor cells and might even be absent^1–4^. Over the intervening decades, we have learned that mitochondria are both present and functional in tumor cells and may in fact play a critical role in cancer development and response to treatment^1–10^. However, our existing understanding of the relationship between mitochondrial activity and tumorigenesis remains mixed, at best.

On one hand, mitochondrial activity appears to be critical to tumor cell survival. This conclusion is supported by the anti-tumor activity of mitochondrial inhibitors and the difficulty associated with generating tumor cells completely void of mitochondria^1,9–12^. On the other hand, high mitochondrial activity and low glycolytic activity are associated with indolent tumor behavior and a relatively favorable response to treatment. Conversely, highly glycolytic tumors have also been found to behave more aggressively across multiple tumor types including OCSCC^8,9,13,14^. These findings are complicated by two experimental limitations. Most functional metabolic studies targeting mitochondria can only be performed in the context of preclinical models^8,9,12–14^. Data regarding mitochondrial activity in patient tumors therefore is largely inferred most often from evaluation of mitochondrial number, appearance, mitochondrial (mt) DNA copy number and integrity. Unfortunately, mitochondrial heteroplasmy makes it nearly impossible to ascertain functional implications of mtDNA mutations and variations in copy number^15,16^.

We previously showed that high glycolytic activity, and impaired mitochondrial respiration are linked to OCSCC development through loss of activity of the tumor suppressor p53^8,9,13,14,17^. At the same time, we demonstrated that inhibition of residual mitochondrial activity resulted in substantial and significant potentiation of chemotherapy and radiation effectiveness in preclinical OCSCC models^8,9,13,14,17^. This paradoxical finding warrants further study for two reasons. First, it is important to understand how mitochondrial activity and tumorigenesis are linked in OCSCC, particularly how the former contributes to the latter. Second, in order to develop effective metabolic strategies, we must better understand the potential impact of mitochondrial targeting in OCSCC. In the current study, we sought to evaluate the relationship between inferred mitochondrial activity and tumorigenesis and treatment response in patients with OCSCC, in order to further contextualize our previous preclinical data. Given the inherent limitations associated with inferring mitochondrial function from analysis of mtDNA we chose to focus on nuclear encoded genes critical to mitochondrial functionality using the OCSCC dataset available in The Cancer Genome Atlas (TCGA). When nuclear encoded mitochondria genes were considered together with genes regulating glycolysis, a metabolic RNA profile was identified that was associated with prognosis in OCSCC and in lung squamous cell carcinoma (LUSC).

## Materials/Subjects and Methods

### Selection of metabolic genes

Genes involved in glycolysis, mitochondrial oxidative phosphorylation, and the pentose phosphate pathway (PPP) were initially manually curated from the literature (Supplementary Tables 1 and 2). Briefly, we focused on 98 genes encoding metabolic enzymes and/or direct regulators of metabolic enzymes. In subsequent analysis, Gene Ontology (GO) search terms glycolysis, glycolysis positive regulation, and oxidative phosphorylation were used to generate a network-based list of 118 genes involved in glycolysis and mitochondrial oxidative phosphorylation, referred to throughout the manuscript as OXPHOG (Supplementary Table 3).

### Analysis of mutations and RNA expression for metabolic genes

We utilized the two largest datasets of squamous cell carcinoma currently available within the TCGA database, specifically the OCSCC and LUSC datasets. A MAF file annotating mutations in the OCSCC TCGA cohort was downloaded from cbioportal (http://www.cbioportal.org/); whereas mutations present in the TCGA LUSC cohort were obtained from Campbell *et al*.^18^. Mutations were considered impactful if their SIFT^19^ score was deleterious or their PolyPhen^20^ score was probably or possibly damaging. RSEM normalized gene expression files, as well as clinical parameters including tissue histology and survival data were downloaded directly from the Broad firehose site (https://gdac.broadinstitute.org/) for all cohorts. RNA-Seq data for noncancerous normal tissues including lung, esophagus, and skeletal muscle were downloaded from the Genotype-Tissue Expression (GTEX) database hosted on the University of California Santa Cruz Xena public data hub (https://xena.ucsc.edu/public-hubs/). Differential expression of the 118 OXPHOG genes between clusters 1 and 2 was analyzed with multiple t-tests using the Benjamini and Hochberg adjustment to control the FDR = 0.05. Chi-square tests to examine possible associations between mutations and patient clusters were performed using Microsoft Excel software (V1903), and adjusted P-values calculated with the Benjamini and Hochberg correction at an FDR = 0.01.

### Survival analysis and hierarchical clustering

Kaplan-Meier curves, median survival (MS) times, and P-values (Log-rank/Mantel-Cox) were generated with GraphPad Prism software (V.7). Hierarchical consensus clustering was based on a re-sampling model previously described by Monti *et al*.^21^ with some modification and selection of optimal cluster numbers (i.e., “c”) based on a novel algorithm we developed using Euclidean distances to measure closeness to theoretical perfection (see Supplementary Methods and Supplementary Fig. 1).

### Single sample gene set enrichment analysis (ssGSEA)

The Broad GenePattern cloud website (https://cloud.genepattern.org/gp/pages/login.jsf) host was used to run ssGSEA, using linear values of RNA expression as inputs, along with gene lists for muscle, hypoxia, or immune subsets (Supplementary Table 4). The hypoxia gene set file was downloaded from the Broad Hallmark pathways lists. Genes elevated in skeletal muscle were downloaded from the human protein Atlas (https://www.proteinatlas.org/humanproteome/tissue/ skeletal+muscle) and manually filtered to 28 genes with at least a 10-fold differential expression between skeletal muscle and maximum expression in 45 different non-muscle normal tissues available from the GTEX database. Gene lists for 15 different immune subsets were obtained from the publication by Senbabaoglu *et al*.^22^ and filtered to remove genes with low specificity for leukocytes as described in Supplementary Methods, and illustrated by Supplementary Fig. 2, and Supplementary Table 5. Differences in ssGSEA values for each immune subset among different patient OXPHOG clusters were examined with a two-way ANOVA followed by a Tukey multiple comparison test with adjusted P values, using GraphPad Prism. To examine relative enrichment of CD8 or cytotoxic cells to Treg, ssGSEA values were transformed into Z scores and the Treg value was subtracted from the CD8 or cytotoxic value for each patient.

## Results

### Metabolically targeted mutational and expression analysis

OCSCC tumors demonstrated a low mutational frequency in core metabolic genes (Supplementary Table 1; Supplementary Fig. 3A) either individually, or when organized by basic metabolic pathway. Metabolic pathway alterations defined by mutation did not significantly impact survival of OCSCC patients (Supplementary Fig. 3B). However, there was a trend towards reduced survival in OCSCC patients with nuclear encoded mitochondrial gene mutations (MS = 20.73 months) compared to those without such alterations (MS = 53.91 months). The same analysis was performed in a separate dataset of LUSC. Overall, mutational frequency of metabolic genes was higher (Supplementary Table 2; Supplementary Fig. 4A) compared to OCSCC tumors; there was no significant correlation between mutations and overall survival (Supplementary Fig. 4B), despite a trend for reduced survival in patients with impactful mutations in nuclear encoded mitochondrial genes. Transketolase like 2 (TKTL2) had a mutational frequency above 5% in LUSC, but failed to reach statistical significance by MutSiq analysis (Q-value= 0.06)^18^ and demonstrated a random missense mutational pattern across the length of the gene (Supplementary Fig. 5), making it unclear whether the gene was a driver or passenger.

We performed unsupervised two-way hierarchical consensus clustering to stratify OCSCC patients based on their expression of nuclear-encoded mitochondrial oxidative phosphorylation (OXPHOS), glycolysis, or pentose phosphate pathway (PPP) genes using TCGA RNA expression data. Using OXPHOS or glycolysis gene expression, 8 and 7 patient clusters were identified (Supplementary Fig. 6A), respectively, based upon optimal NED scores determined from their transformed similarity matrices (Supplementary Fig. 7 and Supplementary Methods). OXPHOS cluster 1 patients had increased expression of OXPHOS genes (i.e., black gene cluster) compared to patient clusters 3–8 (Supplementary Fig. 6A, left) and had significantly better MS than patients in clusters 2–4 (P < 0.04) Supplementary Fig. 6B, left). Patients with lower expression of glycolysis genes in cluster 1 (Supplementary Fig. 6A,B, right panels) also had significantly improved MS (169.2 months) compared to patients in cluster 2 (24.3 months, p < 0.003), or clusters 6 and 7 (54.9 and 29.0 months, p < 0.04). In contrast, clustering by PPP pathway genes (Supplemental Fig. 6A, bottom) revealed no survival differences (not shown). Because our data suggested that downregulation of glycolysis genes and upregulation of OXPHOS genes in tumors led to improved survival, we combined the gene lists and re-clustered OCSCC patients (Supplementary Fig. 8A). As expected, patient cluster 1 with highest expression of mitochondrial genes and reduced expression for a subset of glycolysis genes had significantly better survival compared to patient clusters 2,3, and 4 combined (p < 0.04, Supplementary Fig. 8A, right) with a MS of 169 months compared to 32.6 months.

### Robustness of the metabolic expression phenotype across disease site and gene list

LUSC shares some genomic drivers and pathway alterations with OCSCC that are common to squamous carcinomas^21^. Analysis of the 115 mitochondrial and glycolytic genes in TCGA LUSC patients identified a similar cluster of patients with higher nuclear-encoded mitochondrial gene expression but reduced glycolysis gene expression in their tumors (patient cluster 1, Supplementary Fig. 8B). Cluster 1 LUSC patients also had significantly longer overall MS (103.4 months) compared to other patient clusters. For example, the MS for patient cluster 2 with slightly less mitochondrial gene expression was only 39 months (p < 0.05) and patient cluster 7 with the lowest mitochondrial gene expression but higher glycolysis gene expression had a MS of just 30.6 months (p < 0.002).

**Figure 1:**
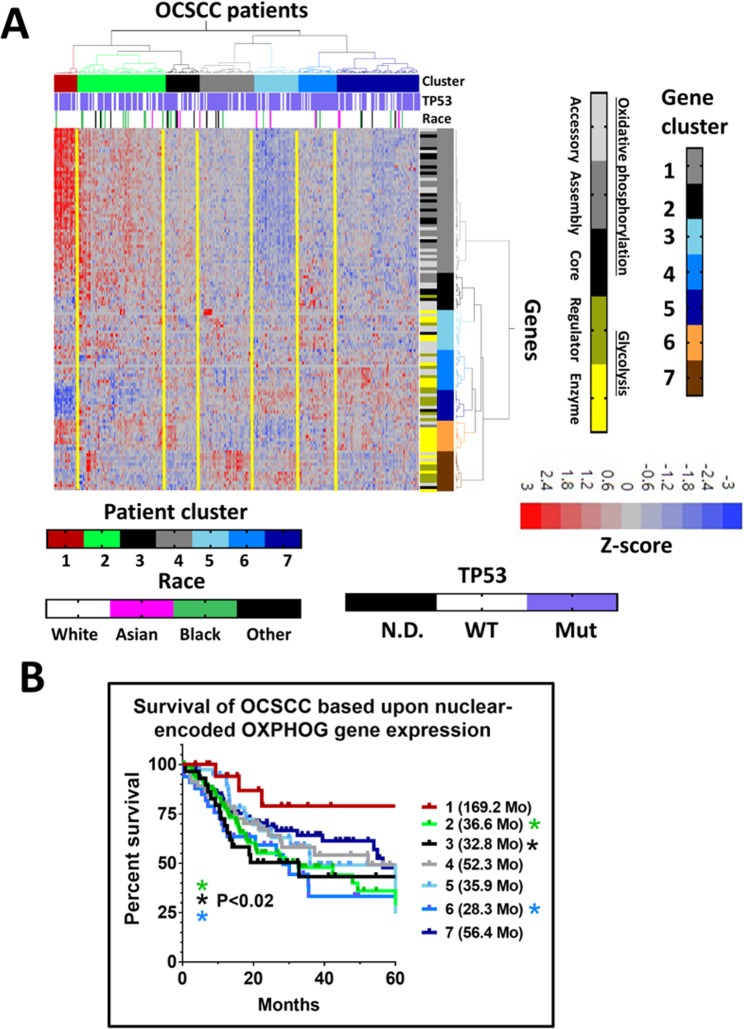
Expression patterns of OXPHOG genes correlate with survival in OCSCC. (**A**) Expression heatmap of 118 OXPHOG genes following two-way unsupervised hierarchical consensus clustering of TCGA OCSCC patients. Race and *TP53* mutational status are annotated horizontally and gene function is annotated vertically according to the legend. (**B**) Kaplan-Meier curves comparing overall survival of patients in the different OXPHOG clusters. P-values reflect comparisons to cluster 1.

Next, we examined if increased survival in patient cluster 1 for both disease sites was highly dependent upon the specific metabolic genes chosen for analysis. Using the Gene Ontology (GO) knowledgebase and GO search terms we generated a second list of 118 genes involved in oxidative phosphorylation and glycolysis (OXPHOG) (Methods, Supplementary Table 3). The OXPHOG gene list derived from GO search terms only partially overlapped with the manually curated genes from our prior analysis (sharing just 65 genes) but included 53 new ones (Supplementary Fig. 9). We performed unsupervised hierarchical consensus clustering of OCSCC tumors with the new OXPHOG gene set (Fig. 1, Supplementary Table 6) and again identified a subset of patients (cluster 1, Fig. 1A) with elevated expression of nuclear-encoded mitochondrial genes (involved in oxidative phosphorylation) that demonstrated improved survival compared to their counterparts (Fig. 1B). The difference in MS for patient cluster 1 (169.2 months) was significant (p < 0.02) when compared to patient cluster 2, (36.6 months), cluster 3 (32.8 months), and cluster 6 (28.3 months). A prominent feature of patient cluster 1 was overexpression of multiple genes involved in oxidative phosphorylation. Patient survival did not have a simple linear relationship to expression levels of oxidative phosphorylation genes. Cluster 1 (highest expression) had the best survival followed by patients in clusters 4 and 7 with intermediate expression; whereas patients with the second highest expression (cluster 2) and the absolute lowest expression (cluster 5) had poor survival suggesting a threshold effect (Supplementary Fig. 10). There was no significant association between OXPHOG clustering and patients’ *TP53*-mutational status or race (Fig. 1A).

**Figure 2:**
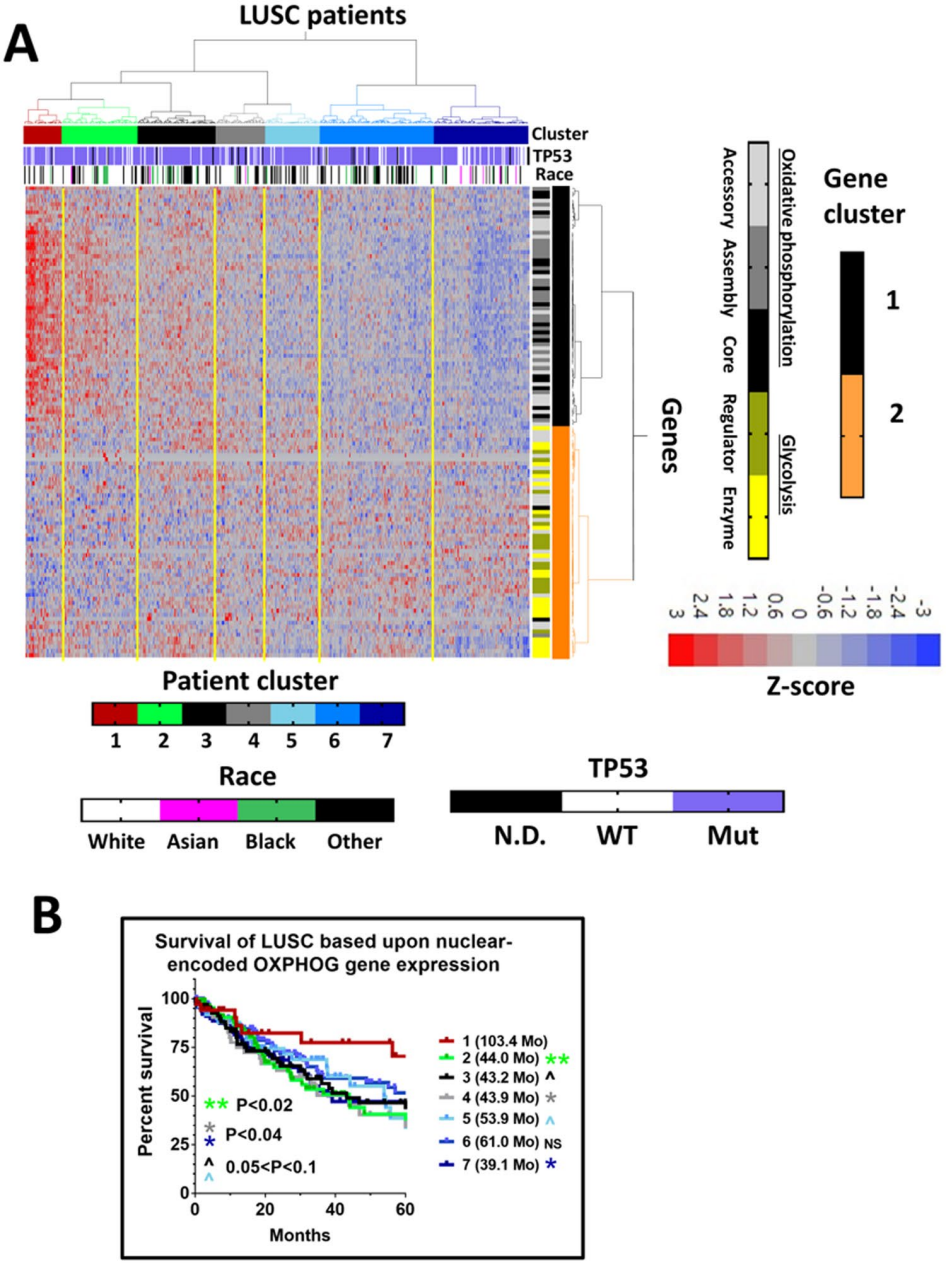
Expression patterns of OXPHOG genes correlate with survival in LUSC. (**A**) Expression heatmap of 118 OXPHOG genes following two-way unsupervised hierarchical consensus clustering of TCGA LUSC patients. Race and *TP53* mutational status are annotated horizontally and gene function is annotated vertically according to the legend. (**B**) Kaplan-Meier curves comparing overall survival of patients in the different OXPHOG clusters. P-values reflect comparisons to cluster 1.

As further validation, we repeated the OXPHOG gene analysis in the LUSC dataset (Fig. 2A). Again, patient cluster 1 had the highest expression levels of genes involved in oxidative phosphorylation and had significantly longer survival times (MS = 103.4 months) compared to other patients (>2 fold, Fig. 2B). The same threshold effect found for OCSCC was evident for LUSC. Patient cluster 2 (second highest oxidative phosphorylation gene expression) and patients with lowest expression (now cluster 7, Fig. 2) had significantly worse MS of just 44 months (p < 0.02) and 39.1 months (p < 0.04), respectively. Similar to OCSCC, LUSC patients with intermediate expression levels of oxidative phosphorylation genes had MS somewhere between these extremes. No significant association was seen between OXPHOG clustering and *TP53*-mutational status or race in LUSC as well. OXPHOG clustering reached statistical significance on univariate analysis of both datasets and approached significance on multivariate analysis in both datasets (Supplementary Tables 8 and 9). The relative effect size of OXPHOG clustering (Exp(B)) was greater than the presence of extranodal extension in OCSCC (one of the most important prognostic indicators for survival in this disease site) and nearly as large as the presence of positive surgical margins in LUSC^23^.

Oropharyngeal squamous cell carcinoma (OPSCC) localized to the base of tongue and tonsillar region is associated with the human papillomavirus (HPV), has distinct biology, and demonstrates considerably better survival compared to stage-matched OCSCC^24–28^. We tested the relationship between the OXPHOG gene set and survival in this subsite of HNSCC. Two-way hierarchical consensus clustering identified a subset of patients with high expression of oxidative phosphorylation genes, but surprisingly these patients had the worst survival in this site even though that they were predominately HPV+ (data not shown). Therefore, we focused our cluster analysis solely on the HPV+ OPSCC patients (Supplementary Fig. 11A). Although only 5 patients were in cluster 1 with higher expression of oxidative phosphorylation genes, 2 patients experienced very early death (<5months) leading to significantly worse MS (p < 0.0001, Supplementary Fig. 11B). Owing to the small number of patients, it is unclear if this was just a statistical anomaly. Consequently, we grouped together all HPV+ OPSCC with OCSCC patients to see where the former would cluster (Supplementary Fig. 12). Most of the HPV+ OPSCC patients with higher oxidative phosphorylation (i.e., OPSCC HPV+ cluster 1 from Supplementary Fig. 11) clustered together with *OCSCC patients* that were originally from patient cluster 2 with poorer survival when just OCSCC was analyzed (Supplementary Fig. 12). However, closer inspection revealed that the major reason HPV+ OPSCC patients did not co-cluster with OCSCC patients in cluster 1 was because of differences in expression of a few glycolysis related genes, rather than because they had lower expression of oxidative phosphorylation genes (fold changes annotated in vertical bar, Supplementary Fig. 12). Therefore, it is difficult to discern if the anomalous results observed for HPV+ OPSCC are due to small sample size or factors associated with the distinct biology of HPV.

### Possible factors contributing to the OXPHOG gene signature

**Figure 3:**
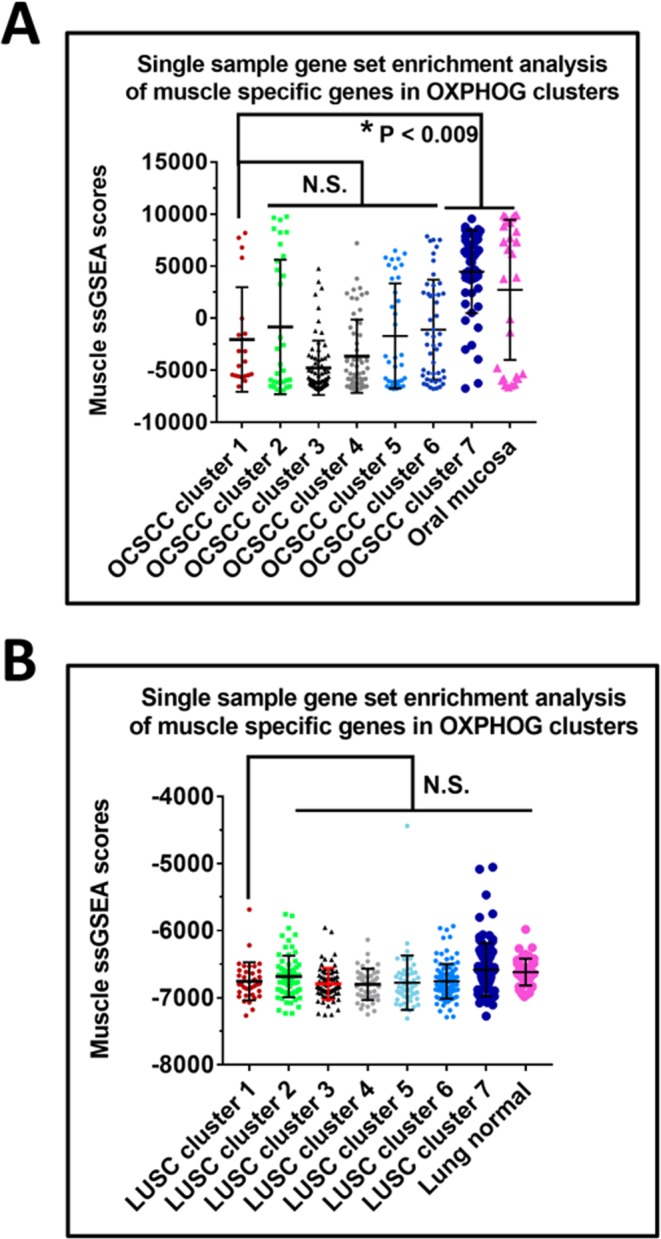
Elevated oxidative phosphorylation gene expression in tumors is not due to muscle contamination. The ssGSEA muscle scores for tumors in each cluster were compared to each other and to adjacent normal for (**A**) OCSCC and (**B**) LUSC. Although muscle contamination was present in some tumors and in normal oral samples, no significant differences were found between tumors in clusters 1, 2, and 3 in either OCSCC (**A**) or LUSC (**B**).

**Figure 4:**
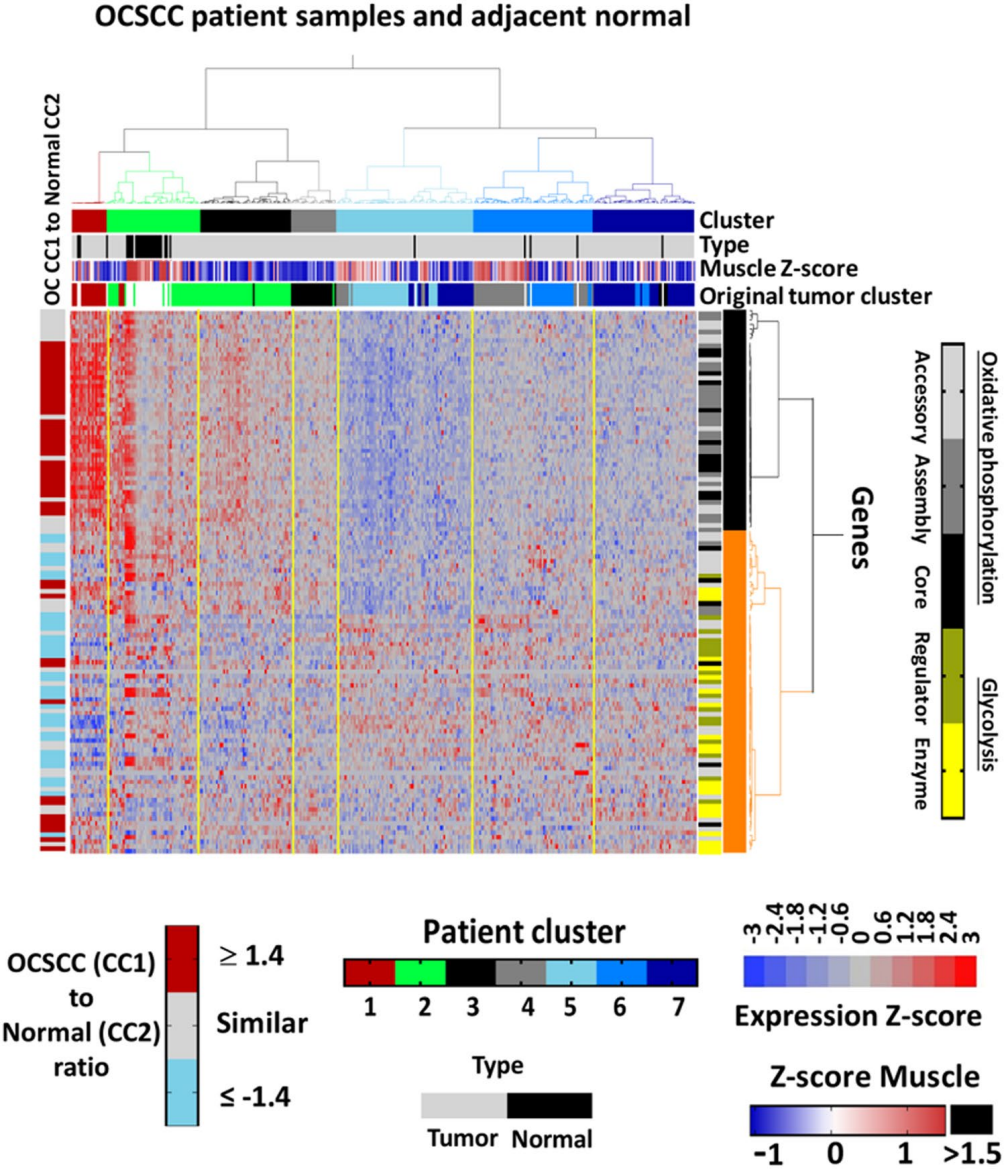
Elevated expression of oxidative phosphorylation genes in cluster 1 represents a super-physiologic state. Expression heatmaps of 118 OXPHOG genes following two-way unsupervised hierarchical consensus clustering of TCGA OCSCC. Normal samples are annotated across the top with black boxes and OCSCC muscle contamination determined from Z-scores of ssGSEA values (i.e., from Supplementary Table 11) are annotated horizontally just below. The original OXPHOG cluster of tumors only is also annotated with colored horizontal boxes, and gene function is annotated vertically according to the legend. The ratio of group geometric averages between tumors in cluster 1 and normal samples in cluster 2 is annotated on the left with a vertical bar according to the legend.

We performed a three-tier analysis of OCSCC and LUSC tumor sets to identify putative explanations for cluster 1 biological and clinical behavior. First, we evaluated for possible contamination of cluster 1 specimens by higher levels of skeletal muscle tissue which might explain differential expression of OXPHOG genes. We utilized a panel of 28 genes with greater expression in normal muscle than 45 other normal tissues (Methods, Supplementary Fig. 13). Neither OCSCC nor LUSC cluster 1 specimens were enriched for skeletal muscle compared to the other clusters (Fig. 3, Supplementary Table 11). Second, we introduced matching normal tissue data into the analysis (Supplementary Tables 6 and 7) to examine if contamination with normal squamous mucosa could be a factor and to better interpret gene expression levels. Patient cluster 1 was not enriched for normal tissue samples in either OCSCC (Fig. 4) or LUSC (Supplementary Fig. 14). The majority of genes with increased expression of RNA (i.e. ≥1.4-fold) in OCSCC compared to normal samples were involved in oxidative phosphorylation, while the majority of genes with decreased expression in OCSCC compared to normal samples (i.e. ≥1.4-fold reduction) were involved in glycolysis (Fig. 4). Collectively, the data demonstrate that OCSCC and LUSC patients in cluster 1 expressed supra-physiological levels of oxidative phosphorylation genes.

**Figure 5:**
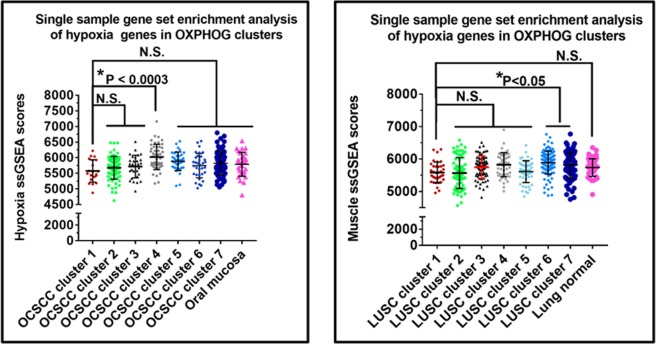
Survival differences between clusters 1 and 2 are unrelated to hypoxia. Levels of hypoxia were estimated by ssGSEA analysis and compared among OXPHOG clusters from OCSCC (**A**) or LUSC (**B**). No statistically significant differences could be found between samples from cluster 1 and 2 in either cancer type.

Third, we evaluated the potential effect of hypoxia on metabolic gene expression using a 200 hypoxia-related gene signature (Supplementary Table 4, Fig. 5, Supplementary Table 11). No significant differences were found in hypoxia scores between OCSCC OXPHOG patient clusters 1,2,3 or 6 that would explain differences in MS. Likewise, no significant differences in hypoxia scores existed between LUSCC OXPHOG patient clusters 1,2, and 4 that could account for the survival differences.

### Mitochondrial copy number

**Figure 6:**
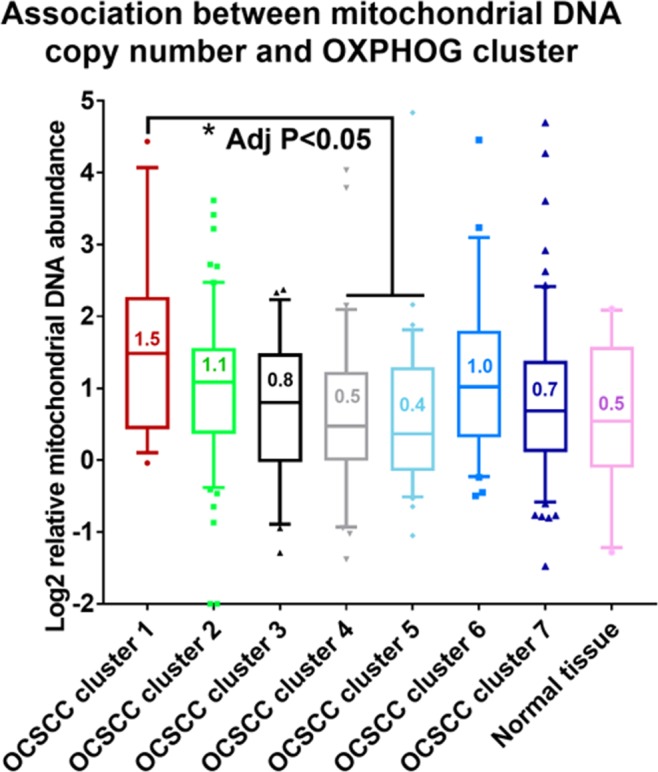
Differences in mitochondrial copy number may be associated with OXPHOG cluster. Published data available for mitochondrial DNA levels in TCGA OCSCC samples (previously estimated through sequencing analysis) were plotted, revealing a trend towards increasing mitochondrial copy number with decreasing OXPHOG cluster number.

Mitochondrial gene expression requires coordination between nuclear encoded components and mitochondrially encoded components. In order to contextualize the OXPHOG signature, we utilized previously published mitochondrial copy number data derived from OCSCC TCGA samples^15^. There was a progression of mitochondrial DNA abundance across the clusters (Fig. 6) with cluster 1 demonstrating the highest abundance, that reached statistical significance for some patient clusters (i.e., p < 0.05 for clusters 3 and 4).

### Analysis of gene expression differences

Earlier analysis of normal tissue suggested that patients in cluster 1 from both OCSCC and LUSC with better survival have supraphysiological expression levels of genes involved in oxidative phosphorylation. Because MS dropped precipitously in the adjacent patient cluster with moderately high expression of the same genes, we quantitatively analyzed the parameters of this threshold effect with respect to the OXPHOG genes. T-tests (FDR controlled, alpha = 0.05) identified 53 genes (i.e., 47 mitochondrial and 6 glycolytic pathway genes) differentially upregulated in OCSCC patient cluster 1 compared to OCSCC patient cluster 2, and 53 genes (i.e., 46 mitochondrial and 7 glycolytic pathway genes) increased in LUSC patient cluster 1 relative to LUSCC patient cluster 2 (Supplementary Fig. 15A, Supplmentary Table 12). A majority of genes (32 out of 35) commonly upregulated in patient cluster 1 in both cancer types were involved in the mitochondrial/oxidative phosphorylation pathway. Considerably fewer genes were downregulated in patient cluster 1, with no common ones between cancer types. More than 75% of the differentially regulated oxidative phosphorylation genes in both cancer types were increased at levels ≥1.4 fold in patient cluster 1 compared to cluster 2 (Supplementary Fig. 15B).

To identify possible candidate genes that may be contributing to the increased number of mitochondria and/or upregulation of mitochondrial genes, we searched for other cellular genes that correlated with expression levels of the oxidative phosphorylation genes from the OXPHOG gene list. This was done by first developing a ssGSEA score that robustly represented key genes in the OXPHOG phenotype based on expression of the 32 mitochondrial pathway genes commonly upregulated in patient cluster 1 from both cancers (Supplementary Table 4). As expected, the OXPHOG ssGSEA scores for the 7 different clusters declined with increasing cluster numbers for both cancers with cluster 1 samples having the highest average ssGSEA values (p < 0.0001, Supplementary Fig. 16A). Next, we interrogated expression from> 20,000 genes from each tumor sample for correlations with the OXPHOG ssGSEA score. Most of the genes highly correlated with OXPHOG ssGSEA for both cancers were involved in mitochondrial function. However, one of the highly correlated genes in common was SSBP1 (single stranded DNA binding protein 1), a key protein involved in mitochondrial DNA replication, biogenesis, and copy number^29–32^, which had a correlation of 0.73 in OCSCC and 0.68 in LUSC (p < 0.0001, Supplementary Fig. 16B).

### Correlation of OXPHOG signature with tumor immune microenvironment (TIME)

Using gene lists (Methods, Supplementary Table 4) previously published to differentiate immune subsets, immune subset ssGSEA scores were calculated for each immune subtype within OCSCC (Supplementary Table 13) or LUSC (Supplementary Table 14) samples^22,33^. Consensus clustering using the ssGSEA scores for 16 different types of leukocytes identified 7 OCSCC immune patient clusters (Supplementary Fig. 17A). The highest MS (156.4 months) was in patient immune cluster 6 (Supplementary Fig. 17B), which had the strongest expression of cytotoxic cells, Th1 cells, and CD56dim cells. Immune clustering in the LUSC cohort also produced similar patient clusters; however, there was no obvious association with MS (Supplementary Fig. 18).

**Figure 7:**
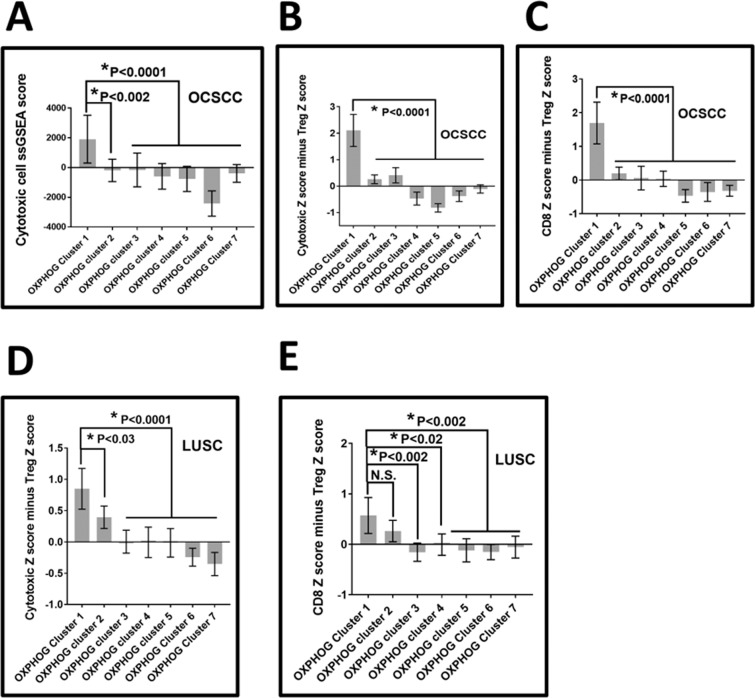
OXPHOG cluster 1 tumors have more favorable baseline tumor immune microenvironments that could explain improved overall survival. Baseline levels of immune subsets present in tumors were estimated through ssGSEA as described in Methods. (**A**) Cytotoxic cells are significantly more abundant in OCSCC tumors from cluster 1 compared to all other clusters. (**B**) The proportion of cytotoxic cells to Tregs modeled through combining variables indicated a significantly more favorable ratio in OCSCC tumors from cluster 1. (**C**) The proportion of CD8 T-cells to Tregs modeled through combining variables indicated a significantly more favorable ratio in OCSCC tumors from cluster 1. (**D**) The proportion of cytotoxic cells to Tregs modeled through combining variables indicated a significantly more favorable ratio in LUSC tumors from cluster 1. (**E**) The proportion of CD8 T-cells to Tregs modeled through combining variables indicated a trend in LUSC toward decreasing ratios with increasing OXPHOG cluster number that reached significance between cluster1 and clusters 3–7.

We used ssGSEA scores for the 16 different leukocyte subtypes to examine if differences in the tumor microenvironment (TIME) could be identified between *OXPHOG patient clusters* (e.g., Figs. 1 and 2) for both OCSCC and LUSC, which might account for differences in survival. In OCSCC, the only significant difference between clusters 1 and 2 was found was for cytotoxic cells (p < 0.0001, Supplementary Table 15), where a 110% decrease in the cytotoxic cell average ssGSEA score was observed for cluster 2 compared to cluster 1 (Fig. 7A). Levels of cytotoxic cells continued to decrease even further among remaining OXPHOG clusters in a gradient-like manner (Fig. 7A).

To examine if the proportion between cytotoxic or CD8+ cells to regulatory T-cells (Treg) could also explain survival differences between OXPHOG clusters in OCSCC, individual ssGSEA values were transformed to Z-scores and variables combined by subtracting the Treg Z score from either the cytotoxic or the CD8+ Z scores for each sample. Differences in these combined Z scores between all clusters were examined by ANOVA and a Tukey multiple comparison test (Supplementary Tables 16 and 17). In OCSCC, OXPHOG cluster 1 patients had significantly higher proportions of cytotoxic to Treg cells (p = 0.0001) and CD8+ to Treg cells (p = 0.0001) than all remaining OXPHOG patient clusters (Fig. 7B,C).

A parallel analysis performed for LUSC failed to detect any statistically significant differences between OXPHOG patients clusters 1 and 2 for any specific leukocyte subtypes; however, there was a trend for decreased cytotoxic cells in cluster 2 that became highly significant for remaining patient clusters (Supplementary Table 18) when compared to OXPHOG cluster 1. Like OCSCC, the proportion of cytotoxic cells to Tregs was significantly higher in cluster 1 compared to cluster 2 (p < 0.03) in LUSCC, which became highly significant compared to remaining LUSC OXPHOG patient clusters (Supplementary Table 19, Fig. 7D). Although the difference between CD8 to Treg proportion between LUSC OXPHOG clusters 1 and 2 did not reach significance (Supplementary Table 20), there was a trend of decreasing values that was significant compared to remaining clusters (Fig. 7E).

### Increased T-cell infiltrate is unlikely to explain higher expression of oxidative phosphorylation genes

**Figure 8:**
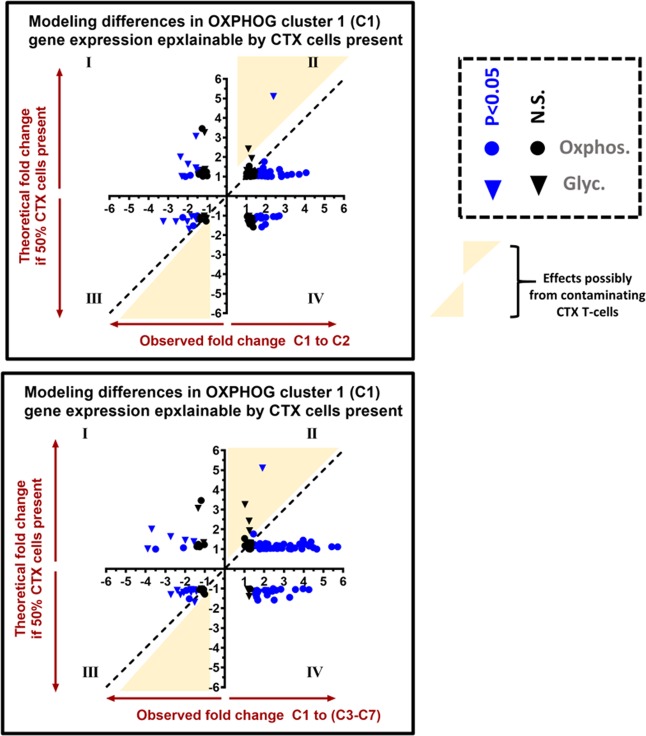
Increased contaminating cytotoxic T-cells are unlikely to account for altered expression of OXPHOG gene levels in cluster 1 tumor samples. The fold-change in geometric means for all 118 OXPHOG genes expected for a mixed theoretical sample consisting of 50% cytotoxic-T cells and 50% tumor cells compared to a theoretical sample of pure tumors, based on single cell RNA-seq data (see methods and results) is plotted on the Y-axis (top and bottom panels). The actual observed ratio of group geometric means from tumors in cluster 1 compared to cluster 2 (top panel) or clusters 3–7 (bottom panel) for all 118 OXPHOG genes was plotted on the X-axis. Ratios for genes with symbols beneath the identity function (dotted line) in quadrants II and IV, or above the identity function in quadrants I and III are highly unlikely to be explained by increased cytotoxic T-cells in cluster 1. Yellow zones in the plot are regions where contaminating T-cells could possibly contribute to differences in gene expression found for patient cluster 1. Blue symbols represent genes that were significantly different in cluster 1 compared to other clusters, and black symbols represent no significant differences in gene expression levels. Circles correspond to genes that function in oxidative phosphorylation, and square symbols represent genes that regulate glycolysis.

To examine whether expression of OXPHOG genes in infiltrating cytotoxic T cells could explain elevated gene expression in OXPHOG patient cluster 1 tumor samples, we leveraged the single cell RNA seq dataset (scRNA-seq) published for HNSCC tumors available online (GSE103322)^34^. First, we split the scRNA samples into either tumor or T-cells based on published annotation. Then we used our previous immune gene list (Supplementary Table 4) to cluster individual T-cell samples and identify cytotoxic T-cell subsets (Supplementary Fig. 19). Next we found the average OXPHOG gene expression for cytotoxic T-cells and tumor cells and modeled expected fold changes to gene expression that would arise solely due to differences in the presence of T-cells under the very conservative assumption that the OXPHOG cluster 1 samples had 50% cytotoxic cell contamination compared to 0% in other clusters. The expected fold-changes for each gene due to presence of T-cells were then plotted against the actual fold changes observed and displayed graphically (Fig. 8). The actual expression levels of oxidative phosphorylation genes were on average >2 fold higher in OXPHOG cluster 1 patients compared to other patient clusters; whereas expression levels of glycolysis genes in OXPHOG cluster 1 showed the opposite trend and were on average at least 1.5-fold lower (Fig. 8). The majority of significant changes in OXPHOG gene expression found in OCSCC patient cluster 1 would not be explainable by differences in the presence of cytotoxic T-cells (i.e. yellow zones, Fig. 8).

### Correlation of genotype with OXPHOG phenotype

We analyzed whether there was a difference in distribution of the 48 driver mutations identified for HNSCC or the 22 driver mutations found in LUSC among clusters 1 and 2. A slight enrichment for increased mutations in KDM6a and KMT2B was found in OCSCC cluster 1 (Supplementary Table 20), but none of the other driver genes in either OCSCC or LUSC (not shown) had significantly different mutation frequencies between OXPHOG cluster 1 and other clusters (Supplementary Tables 21 and 22).

## Discussion

Ever since Warburg first described a differential metabolic phenotype in cancer cells, scientists have hoped to translate this basic fact of cancer biology into viable therapeutic strategies. The natural starting point was to inhibit tumor glycolytic activity, while minimizing normal tissue toxicity^3,35^. Despite extensive pre-clinical and clinical work, glycolytic targeting has failed to translate into clinical practice for the majority of solid tumors including SCC^8,9,36–38^. Over the last decade, our group along with other investigators have shifted focus toward modulation of mitochondrial activity to generate *de novo* anti-tumor activity, or to potentiate chemo-radiation effects in solid tumors^10,13,39,40^. Although supported by preclinical studies and retrospective clinical data, inhibition of mitochondrial respiration to improve treatment response is somewhat counterintuitive, since high levels of mitochondrial activity are generally linked to improved clinical outcomes and indolent tumor behavior^9,14–16,39,41–43^.

Our study highlights the fact that the role of energy metabolism and mitochondrial function in cancer biology and response is complex. We found that OCSCC and LUSCC mutations in metabolic genes encoding enzymes and proteins involved in either glycolysis or oxidative phosphorylation are infrequent and likely to be random, unlike in other tumor types^4,11,44^. However, we did observe a strong association between expression levels of a subset of oxidative phosphorylation genes and patient outcomes in two independent cohorts of SCC, which included OCSCC and LUSC. Very high expression levels of genes encoding core proteins from all four ETC complexes as well as regulatory and structural proteins involved in oxidative phosphorylation identified subsets of OCSCC and LUSC patients with significantly better survival. Interestingly, patient survival did not have a simple linear relationship to expression levels of oxidative phosphorylation genes but varied predictably according to distinct thresholds.

Previous investigations have linked mitochondrial copy number to cancer (non-SCC) survival^15^. We found a clear trend of increasing mitochondrial copy number with increasing expression of oxidative phosphorylation genes, although differences only reached statistical significance for OCSCC cluster 5 which had roughly *three times less* mitochondria than cluster 1, the lowest expression of oxidative phosphorylation genes, and poor survival. Consistent with the role of increased mitochondrial copy number as a driver of differences in nuclear encoded OXPHOG genes, we found expression of SSBP1—which regulates mitochondrial biogenesis and copy number—to be highly correlated with the OXPHOG gene signature in both OCSCC and LUSC. The threshold effect we observed with respect to OXPHOG genes, MS, and associated mitochondrial copy number could easily explain why past studies may not agree regarding the prognostic significance of mitochondrial levels in tumors.

At present it is unknown exactly why tumors in cluster 1 had the best prognosis. It has been speculated that indolent tumors whose metabolic phenotype remains closer to that of normal tissue may display a less aggressive behavior. Our data do not support this model as normal tissue from lung and the oral cavity had substantially lower OXPHOG gene expression, except for the normal oral samples with evidence of high muscle contamination. However, cluster 1 tumors showed no elevated muscle contamination arguing mitochondrial associated genes were at super-physiological levels compared to normal squamous mucosa. This is further supported by the much lower mitochondrial DNA levels in the normal tissue. Moreover, no differences in normoxia as estimated by the hypoxia gene signature were found between clusters 1,2, or 3 that would account for better survival in cluster 1.

Tumor metabolic activity has previously been shown to be a powerful driver of immune infiltration, primarily through differential production of lactate, a critical suppressor of leukocyte differentiation and activity^45,46^. It is intriguing that that expression of OXPHOG genes generated a differential tumor immune microenvironment (TIME) imputed from gene expression analysis. An increase in cytotoxic and CD8+ cells observed in OCSCC would be consistent with an improvement in patient survival based on our understanding of TIME effects in SCC^47^. Furthermore in both OCSCC and LUSC, the ratio of cytotoxic cells to Treg cells was most favorable in cluster 1 tumors and declined with increasing cluster number supporting the idea that the metabolic environment of the tumor can functionally impact the immunosuppressive nature of the TIME. If the metabolic profile of cluster 1 tumors is permissive for a preferentially immunoreactive TIME, it would represent an exciting finding. To date, there is no clear explanation for differential TIME across SCC and there is no consistently applicable biomarker of effectiveness for existing immunomodulatory agents.

There are obvious limitations to our study as gene expression may not completely reflect protein levels or enzymatic activity. However, the data summarized in the current study strongly suggest that nuclear encoded mitochondrial genes may indeed be a good surrogate and that mitochondrial function and glucose utilization plays a very complex role in SCC tumorigenesis and treatment response. Therefore, targeting of mitochondrial activity should be carefully considered from multiple perspectives.

## Supplementary information

Supplementary figures and methods.

Supplementary tables.
