# Supplementary material for: High expression of oxidative phosphorylation genes predicts improved survival in squamous cell carcinomas of the head and neck and lung
Source: Sci Rep. 2020 Apr 14;10:6380. doi: 10.1038/s41598-020-63448-z (PMC7156383; doi:10.1038/s41598-020-63448-z)
Supplement: Supplementary file 1 — Supplementary figures and methods. [file 41598_2020_63448_MOESM1_ESM.pdf]

High expression of oxidative phosphorylation genes predicts improved survival in squamous cell carcinomas of the head and neck and lung.

Mitchell Frederick [1], Heath D. Skinner [2], Sawad A. Kazi [1,3]Andrew G. Sikora [1], Vlad C. Sandulache [1]

Author's Affiliations:

1- Bobby R. Alford Department of Otolaryngology Head and Neck Surgery, Baylor College of Medicine, Houston, TX

2- Department of Radiation Oncology, UPMC Hillman Cancer Center, Pittsburgh, PA

3- School of Natural Sciences, University of Texas, Austin, TX

Conflict of interest: the authors have no conflict of interests to disclose

Financial support: This research was supported in part by Institutional Start-up Funds (VCS, MF) and the Mike Hogg Fund (VCS). VCS is supported by National Institute of Dental and Craniofacial Research (NIDCR) R03DE028858 and a Translational Innovator Award from the American Head and Neck Society and the American Academy of Otolaryngology – Head and Neck Surgery Foundation. Additional support for HDS from The Cancer Prevention & Research Institute of Texas (CPRIT) RP150293 and the National Cancer Institute (NCI) R01CA168485.

The content is solely the responsibility of the authors and does not necessarily represent the official views of the National Cancer Institute or the National Institutes of Health. The funders had no role in study design, data collection and analysis, decision to publish, or preparation of the manuscript.

Running Title: Oxidative phosphorylation gene expression predicts survival.

Keywords: oxidative phosphorylation, p53, mitochondria, oral cavity cancer, lung cancer

Corresponding author:

Vlad C. Sandulache MD PhD

Bobby R. Alford Department of Otolaryngology – Head and Neck Surgery

Baylor College of Medicine

One Baylor Plaza, MS: NA102

Houston, TX 77030

Phone: (713) 798-7218

Fax: (713) 798-5078

E-mail: vlad.sandulache@bcm.edu

Supplementary Methods

Hierarchical consensus clustering: We used a modification of the resampling-based consensus clustering technique published by Monti et el. [21] Code was written in Python (see below) to adapt the consensus clustering scheme to Ward's method of agglomerative hierarchical clustering. Essentially, "N" number of input samples are randomly resampled by taking a fixed proportion "p" of starting samples and individually running Ward's clustering for "K" number of resamplings. After each individual clustering run, the dendrogram is divided into increasing numbers of clusters ranging from 2 to "c", where the input "c" corresponds to the maximum number of cluster divisions considered. For a given value of "c" the program then calculates the total fraction of times that any two samples are grouped together in the same cluster over K resamplings. The matrix of co-localization proportions for each value of "c" is referred to as the similarity matrix. For each "c" the similarity matrix is used as the final input to then perform Ward's hierarchical agglomerative clustering to produce the consensus cluster groupings with the same choice of "c" clusters. Thus, a different similarity matrix is produced for each choice of cluster numbers ranging from 2 to "c". For the analyses in this manuscript, K=300, p=0.8, and c was set to a maximum of 15. For consensus clustering of the second dimension (e.g., genes or features), sample-wise Z scores for each feature were first calculated before transposing the input table, and then the same consensus clustering algorithm was performed independently to find the optimal number of clusters for features. Dendrograms created in JMP using consensus matrix values from the optimal cluster choices (described below) were combined with Z-score heatmaps (samples along the horizontal axis and features across the vertical axis) to generate the final heatmap figures.

Optimal choice of cluster number: A method for choosing optimal cluster numbers has been described[21] however; it still requires visual inspection. We approached the problem with a novel calculus (See Supplementary Figure 1). An optimal choice of "c" clusters is the one that generates the best similarity matrix. The ideal similarity matrix ordered horizontally and vertically according to clusters should have

diagonal blocks of values equal to 1 within clusters and equal to zero outside of clusters because elements of the cluster should group together 100% of the time during resampling but 0% of the time with outside elements. However, the ideal consensus cluster matrix will have different numbers of “1s” and zeros depending upon the number of clusters chosen.

To overcome this limitation, the similarity matrix is transformed so that values outside the cluster blocks are defined as the probabilistic compliment or 1 minus the existing value. This redefines values outside the cluster blocks as the proportion of times the elements do not cluster together, which theoretically should be 100% or a value of 1. Now, the ideal transformed consensus matrix has values of 1 in every cell regardless of how many clusters are chosen and comparisons can be made across choices of “c” number of clusters The goodness of the cluster choice can be taken as the deviation of the transformed consensus matrix from the ideal transformed consensus matrix (i.e. the identity matrix), by calculating the Euclidean distance in matrix space (Supplementary Figure 1). Because the Euclidean distances will be proportionate to the square root of the total sum of squares in the formula (equal to N^2), the distances can be normalized by dividing by the number of samples in a cohort to yield a Normalized Euclidean Distance—making it possible to compare metric across different cohorts with differing numbers of starting samples. In practice, the NED will fluctuate with the choice of “c” number of clusters but theoretically will approach a value equal to zero when the number of clusters is equal to the number of samples. However, there is a price to be paid for choosing too many clusters, resulting in a loss of information about relatedness that defeats the original purpose. Therefore, the best practice is to plot the NED versus number of “c” chosen clusters and identify local minima, so that both NED and number of clusters is simultaneously optimized. An example pot of NED for data in this paper are depicted in Supplementary Figure 7.

Immune subset gene lists: We began with a published list of genes previously used to distinguish leukocyte subsets for ssGSEA[22] and further filtered the list by vetting genes based on their cross-correlation. Genes that are unique for immune subsets and truly specific for leukocytes should be tightly

correlated. Cross-correlation coefficients of genes in pooled RNA-Seq data from the HNSCC and LUSC cohorts were clustered and heat maps generated with JMP software to identify genes that were highly correlated (e.g., Supplementary Figure 2) for each immune subset. For genes to be selected, the geometric mean of their correlations with other genes defining the immune subset had to be ≥ 0.3 (e.g., Supplementary Table 5). The final filtered list generated for 16 different immune subtypes is provided in Supplementary Table 4.

Python code for agglomerative hierarchical consensus clustering by Wards method:

```
import pandas as pd
import numpy as np
import random, math, time, openpyxl
from sklearn.cluster import AgglomerativeClustering
from openpyxl.styles import PatternFill
from scipy.stats import zscore
from scipy.spatial.distance import euclidean
# VARIABLES TO EDIT PER RUN
# enclose these variables in quotes
path = "C:\\”your path”\\”file name”.xlsx" # directory of input file (double
backslash (\\)
# single backslash (\) | periods (.) in excel file name are invalid
sheet = "Sheet1" # name of the sheet containing the matrix input
sample_column = 'samples' # name of sample column (exactly what is contained in cell
A1) | if noth
# enter "samples" (without quotes) in A1 and set this variable to "samples" (with
quotes)
# do not use quotes for these variables
c = 8 # clusters
k = 300 # resamples
p = 0.8 # resample proportion
t = 50 # number of rows by which time increments will be reported
z = True # True = calculate z scores treating vertical columns as separate datasets
(running as gen
# False = do not calculate z scores (running as features, data set must already be in
z
start_Time = time.time()
inputDF = pd.read_excel(path, sheet_name=sheet)
inputDF.set_index([sample_column], inplace=True)
n = len(inputDF.index)
fileName = path.split('\\')[-1].split('.')[0]
writer = pd.ExcelWriter(fileName + '_consensusClusters.xlsx', engine='xlsxwriter')
transWriter = pd.ExcelWriter(fileName + '_transformedMatrices.xlsx',
engine='xlsxwriter')
resWriter = pd.ExcelWriter(fileName + '_resampledClusters.xlsx', engine='xlsxwriter')
simWriter = pd.ExcelWriter(fileName + '_similarityMatrices.xlsx',
engine='xlsxwriter')
def intersection(a1, a2):
```

```
overlap = 0
for x in range(k):
if np.isnan(a1[x]) or np.isnan(a2[x]):
continue
else:
overlap += 1
return overlap if overlap else 1
1
#creating resampled populations
resampled_Population = [[False for x in range(n)]for y in range(k)]
for r in range(k):
randInts = random.sample(range(n), math.floor(p * n))
randInts.sort()
for i in randInts:
resampled_Population[r][i] = True
for nc in range(2, c + 1):
cluster_increment = time.time()
# constructing intermediate matrix of resampled clusters
wha_Clustering = AgglomerativeClustering(n_clusters=nc, affinity='euclidean',
linkage='ward')
resampled_Clusters = pd.DataFrame(index=inputDF.index, columns=range(0,k))
for s in range(k):
if z == True:
clust = wha_Clustering.fit_predict(zscore(inputDF.iloc[resampled_Population[s]],
axis=0
else:
clust = wha_Clustering.fit_predict(inputDF.iloc[resampled_Population[s]])
resamples = [x for x in range(n) if resampled_Population[s][x] is True] # 1d array
transla
for i in range(len(resamples)):
resampled_Clusters.iloc[resamples[i], s] = clust[i]
resampled_Clusters.to_excel(resWriter, sheet_name='n=' + str(nc))
print('\nFinished constructing intermediate matrix n=' + str(nc))
# constructing similarity matrix from resampled clusters
print('Starting calculation of consensus matrix n=' + str(nc))
simMatrix = pd.DataFrame([[0] * n] * n, columns=inputDF.index, index=inputDF.index)
row_increment = time.time()
for r in range(n):
for c in range(n):
simMatrix.iloc[r, c] += round(
np.sum(np.equal(resampled_Clusters.iloc[r], resampled_Clusters.iloc[c]))
/ intersection(resampled_Clusters.iloc[r], resampled_Clusters.iloc[c]), 4)
if r % t == 0 and r != 0:
print('Finished calculating up to row ' + str(r) + ' in ' + str(time.time() -
row_incre
row_increment = time.time()
simMatrix.to_excel(simWriter, sheet_name='n=' + str(nc))
# sorting simMatrix by Ward clustering
clust = wha_Clustering.fit_predict(simMatrix)
simMatrix['newClusters'] = clust
simMatrix.sort_values(by=['newClusters'], axis=0, ascending=True, inplace=True)
newClust = simMatrix['newClusters']
simMatrix.drop(['newClusters'], axis=1, inplace=True)
clust_df = pd.DataFrame(clust, index=simMatrix.columns, columns=['newClusters'])
clust_df = clust_df.transpose()
```

```
simMatrix = simMatrix.append(clust_df, ignore_index=False)
simMatrix.sort_values(by=['newClusters'], axis=1, ascending=True, inplace=True)
simMatrix['newClusters'] = newClust
2
simMatrix.to_excel(writer, sheet_name='n=' + str(nc), float_format='%.4f')
# calculating probabilistic complements outside cluster blocks
for r in range(n):
for c in range(n):
if simMatrix.iloc[r, -1] != simMatrix.iloc[-1, c]:
simMatrix.iloc[r, c] = 1 - simMatrix.iloc[r, c]
# calculating Euclidean distances
simMatrix['Euclidean dist'] = euclidean(np.array([1] * (n ** 2)),
simMatrix.iloc[:n, :n].to_numpy().reshape(1, n ** 2))
simMatrix.to_excel(transWriter, sheet_name='n=' + str(nc), float_format='%.4f')
print('Finished calculating consensus matrix n=' + str(nc) + ' in ' + str(time.time()
- cluster
del resampled_Clusters, simMatrix, clust_df, newClust, clust, wha_Clustering
writer.save()
transWriter.save()
resWriter.save()
simWriter.save()
print(str(time.time() - start_Time) + ' secs elapsed total')
```

Supplementary Figure Legends

Supplementary Figure 1. Method to calculate normalized Euclidean distance from a similarity matrix to inform optimal cluster choice. (A) Mock similarity matrix output of the type generated from consensus clustering (See Supplementary Methods) for an artificial data set with 9 samples labeled "A"to"H" (left panel). In this theoretical example, the number of clusters "c" after each re-sampling was set to equal 3 to count the number of times any two samples end up in the same cluster. In practice, the procedure is repeated for different values of "c" over a chosen range (e.g., 2 ≤ c ≤ 15) with the desired number of re-samplings "K", but keeping the same re-sampling input sets common across different values of c. Values in the matrix values represent the fraction of times two samples co-cluster together for a given choice of c. The similarity matrix values themselves are subsequently used for clustering to generate "c" number of consensus clusters (e.g., pink, grey, and light blue). The heatmap and cluster tree for the example consensus clusters (A,C, B; D,E; and F,I,G, H) is pictured in the right panel. (B) The similarity matrix is then transformed, so that values outside the consensus clusters (e.g., colored diagonal blocks) are substituted with the probabilistic complement or fraction of times two samples do not co-occur in the same cluster (i.e., 1-similarity matrix value) to yield a transformed similarity matrix (left panel), also represented as a heatmap (right panel). (C) If consensus clustering were perfect, the theoretical transformed similarity matrix would have values of 1 in every cell because samples in the same cluster should always co-cluster together and never cluster with outside members, 100% of the time. (D) Formula for normalized Euclidean distance in matrix space, which defines how far apart the observed transformed matrix for a given value of c is from theoretical perfection or the identity matrix.

Supplementary Figure 2. Heatmap for gene expression cross correlation coefficients among the 15 genes previously published to define cytotoxic cells. RNA expression from the TCGA OCSCC and LUSC tumor cohorts was pooled and cross correlation coefficients calculated among all 15 candidate genes. Correlations were then analyzed by hierarchical clustering to depict the subset of genes best correlating. Genes that clustered together graphically with good correlation were further analyzed as described in the methods to identify the subset of genes with correlations ≥ 0.3 (e.g., Supplementary Tables 4 and 5).

Supplementary Figure 3. Mutations in metabolic genes are infrequent and do not correlate with survival in OCSCC. Somatic mutations in glycolysis (A) or pentose phosphate pathway (B) genes were identified from the OCSCC TCGA summary MAF file. (B) Stratification of patients based upon presence or absence of mutations in glycolysis (top left), pentose phosphate (top right), or nuclear encoded mitochondrial (bottom left) genes did not reveal an association with overall survival, regardless of whether all mutations were considered (blue lines) or just mutations predicted to be impactful (redlines) through SIFT or PolyPhen scores. For survival analysis, all non-synonymous mutations listed in Supplementary Table 1 were used.

Supplementary Figure 4. Mutations in most metabolic genes are infrequent and do not correlate with survival in LUSC. (A) Somatic mutations in glycolysis (top left), pentose phosphate (top right), or nuclear encoded mitochondrial (bottom left) pathway genes were identified from the LUSC TCGA summary MAF file. Only genes mutated with frequency > 1% are graphed. (B) Stratification of LUSC patients based upon presence or absence of mutations in glycolysis (top left), pentose phosphate (top right), or nuclear encoded mitochondrial (bottom left) genes did not reveal an association with overall survival, regardless of whether all mutations were considered (blue lines) or just mutations predicted to be impactful (redlines) through SIFT or PolyPhen scores. For survival analysis, all non-synonymous mutations listed in Supplementary Table 2 were used.

Supplementary figure 5. Random distribution pattern of TKTL2 missense mutations in LUSC patients does not fit the pattern of a cancer driver.

Supplementary figure 6. Link between expression levels of metabolic genes and survival in OCSCC. (A) Heatmaps representing unsupervised hierarchical consensus clustering of patient tumors based on expression of nuclear-encoded mitochondrial pathway genes (top left), glycolysis genes (top right), and pentose phosphate pathway genes (bottom left). (B) Patients in cluster 1 with highest expression of mitochondrial pathway genes had significantly better survival than patients in clusters 2, 3 and 4 grouped together. In contrast, patients in clusters 6 and 7 with highest glycolysis pathway gene expression(right) had significantly worse survival compared to patients in cluster 1. None of the survival differences for patients clustered by pentose phosphate pathway gene expression approached significance. P-values were calculated in comparison to cluster 1 for all graphs.

Supplementary Figure 7. Graphical approach to identify optimal values for "c" (i.e., number of consensus clusters) using nuclear encoded mitochondrial pathway gene expression data from the OCSCC cohort. (A) Normalized Euclidean distances (NEDs) for transformed similarity matrices plotted over a range of possible consensus cluster values following hierarchical consensus clustering of OCSCC patient samples. Red symbols indicate local minima and acceptable choices for number of consensus clusters, while the blue symbol represents the number of clusters actually used for the survival analysis in the text because it corresponds to a good trade off between NED minima and loss of information from too many clusters. (B) Plot of NEDs for transformed similarity matrices plotted over a range of possible consensus clusters following hierarchical consensus clustering of the second dimension (genes). The optimal number of consensus clusters for genes is 5 (blue symbol). For more details see Supplementary Methods.

Supplementary figure 8. A composite list of metabolic genes involved in mitochondrial oxidative phosphorylation and cellular glycolysis identifies a cluster of patients with good prognosis in OCSCC and LUSC. (A) OCSCC patients were clustered based upon combined expression pattern of manually curated metabolic genes from Supplementary Tables 1 and 2, which included 36 glycolysis pathway members and 89 nuclear encoded mitochondrial genes involved in oxidative phosphorylation. Patients in cluster 1 with high expression of oxidative phosphorylation genes and low expression of glycolysis pathway genes had very high survival compared to patients in clusters 2,3, and 4. (B). A similar pattern of patient clusters was found for LUSC where patient cluster 1 also had improved survival relative to other clusters. P-values for Kaplan-Meier curves from both cancers represent comparisons between cluster 1 and other clusters.

Supplementary Figure 9. Overlap of metabolic genes from the manually curated lists and the 118 OXPHOG gene list generated from GO search terms. A Venn diagram illustrates that only 20/36 manually curated glycolysis genes and 45/79 manually curated oxidative phosphorylation genes (OXPHOS =mitochondrial pathway genes) were in common with the 118 GO gene list of OXPHOG genes that also included 53 unique genes.

Supplementary Figure 10. Schematic of the threshold relationship between gene expression and MS in OCSCC and LUSC. Patients with the absolute highest expression levels of oxidative phosphorylation genes had relatively good MS; whereas patients with the second highest gene expression levels did very poorly. Likewise, patients with the lowest expression of oxidative phosphorylation genes also did poorly, but patients with intermediate expression had more favorable MS.

Supplementary Figure 11. High expression of oxidative phosphorylation genes does not improve survival in the context of HPV+ OPSCC. A) Expression heatmap of 118 OXPHOG genes following two-way unsupervised hierarchical consensus clustering of TCGA HPV+ OPSCC patients. Gene function is annotated vertically according to the legend. B) Kaplan-Meier curves comparing overall survival of patients in the different OXPHOG clusters, where patients with the highest expression of oxidative phosphorylation genes (i.e., cluster 1) now had the worst MS. P-value reflects comparisons to cluster 1.

Supplementary Figure 12. Comparison of OXPHOG patient clusters in OSCC and HPV+ OPSCC patients. Pooled unsupervised hierarchical consensus clustering of tumor samples from both subsites was performed. Original cluster assignments for samples when just OCSCC (e.g., Figure 1) or just HPV+OPSCC (e.g., Supplementary Figure 11) were considered are annotated beneath the samples (e.g., red boxes were originally in cluster 1). The new pooled cluster 1 is composed almost exclusively of OCSCC samples, with most HPV+ OPSCC samples originating from OPSCC cluster 1 now associated with OCSCC samples originally from cluster 2. The ratio of geometric means between OCSCC samples originally in cluster 1 and HPV+ OPSCC tumors originally in cluster 1 (but now in pooled cluster 2) is annotated vertically. Despite being grouped in distinct clusters when considered together, OCSCC cluster 1 tumors do not differ that much from HPV+OPSCC tumors originally found in cluster 1 when the sub-sites are clustered separately.

Supplementary Figure 13. Validation of the muscle-specific gene list. The 32 muscle specific genes were used in a ssGSEA of RNA expression from normal skeletal muscle, esophageal mucosa, and normal lung samples available in the GTEX database. As expected, ssGSEA scores from muscle tissue are significantly higher ($P<0.0001$) than the other normal samples by several orders of magnitude.

Supplementary Figure 14. Elevated expression of oxidative phosphorylation genes in cluster 1 represents a super-physiologic state in LUSC. Expression heatmap of 118 OXPHOG genes following two-way unsupervised hierarchical consensus clustering of TCGA LUSC and adjacent normal samples. Normal samples are annotated across the top (e.g., black boxes), along with original tumor cluster number and Z-scores for muscle ssGSEA values. Gene function is annotated vertically according to the legend.

Supplementary Figure 15. Differential expression of OXPHOG genes (DEG) between patient clusters 1 and 2. Multiple T-tests corrected for FDR (alpha =0.05) identified genes with significantly different expression levels between OXPHOG patient clusters 1 and 2 in OCSCC and LUSC. (A) Venn diagram showing overlap of OXPHOG genes significantly upregulated (left panel) or downregulated (right panel) in patient cluster 1 vs cluster 2 for both cancer types, broken down by mitochondrial function (i.e., oxidative phosphorylation) or glycolysis pathway. (B) Histogram depicting average linear fold change of nuclear encoded mitochondrial genes significantly upregulated in patient cluster 1 from OCSCC (left panel) or LUSC (right panel).

Supplementary Figure 16. SSBP1 RNA expression correlates highly with expression of oxidative phosphorylation genes from the OXPHOG list. (A) An oxidative phosphorylation gene list was created from the 32 genes commonly upregulated in patient cluster 1 from both cancer types (e.g., Supplementary Figure 15 and Supplementary Table 12) and used to generate OXPHOG ssGSEA scores for OCSCC (left panel) or LUSC (right panel) samples that were then plotted as a function of previously determined OXPHOG clusters. OXPHOG ssGSEA scores for patients from cluster 1 were significantly elevated in both cancers. (B) High correlation between SSBP1 expression and OXPHOG ssGSEA score across OCSCC (left panel) and LUSC tumors (right panel).

Supplementary Figure 17. Analysis of TIME, OXPHOG gene expression, and survival in OCSCC. (A) Two-way unsupervised hierarchical consensus clustering of immune subset scores defines 7 patient clusters. Immune clusters 4 and 6 show enrichment for OXPHOG cluster 1 patients (annotated across top), where the ratio of ssGSEA scores for more cytotoxic cells (orange gene cluster) to T-regs appears elevated. (B) Patients in immune cluster 6, characterized by relatively high cytotoxic cell subsets, had improved MS (156 months) compared to patients from some of the other immune clusters.

Supplementary Figure 18. Analysis of TIME, OXPHOG gene expression, and survival in LUSC. (A) Two-way unsupervised hierarchical consensus clustering of immune subset scores defines 7 patient clusters. No gross trends are evident between immune cluster, OXPHOG patient cluster, or survival in LUSC.

Supplementary Figure 19. Identification of CD8+ and cytotoxic T-cell subsets from a published cohort of HNSCC scRNA sequencing. Unsupervised hierarchical consensus clustering of T cell samples using expression values of gene lists for Treg, T-cell, CTX cells, CD8+ cells, and other T-cells (e.g. Supplementary Table 4) identified 4 main cell type clusters used to identify samples representing CD8+ or CTX cells. Expression data from the identified cell types was used for the ratiometric gene expression modeling depicted in Figure 8.

A

	A	C	B	D	E	F	I	G	H
A	1	0.8	0.76	0.2	0.3	0.3	0.2	0.25	0.4
C	0.8	1	0.7	0.3	0.2	0.2	0.1	0.3	0.4
B	0.76	0.7	1	0.1	0.3	0.1	0.4	0.18	0.25
D	0.2	0.3	0.1	1	0.8	0.3	0.1	0.2	0.2
E	0.3	0.2	0.3	0.8	1	0.2	0.25	0.4	0.15
F	0.3	0.2	0.1	0.3	0.2	1	0.9	0.65	0.7
I	0.2	0.1	0.4	0.1	0.25	0.9	1	0.75	0.75
G	0.25	0.3	0.18	0.2	0.4	0.65	0.75	1	0.64
H	0.4	0.4	0.25	0.2	0.15	0.7	0.75	0.64	1

Example of similarity matrix For c = 3 clusters

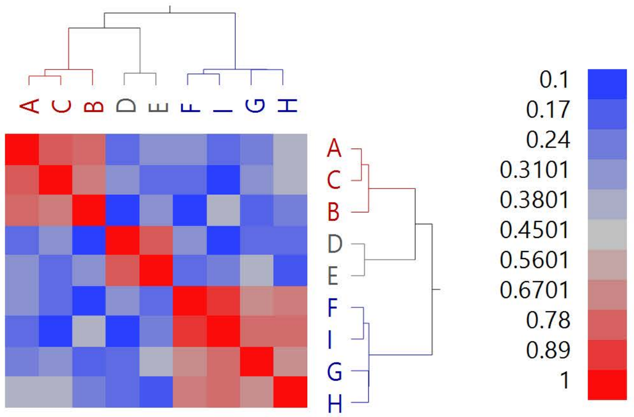

Consensus cluster of samples c = 3

B

	A	C	B	D	E	F	I	G	H
A	1	0.8	0.76	0.8	0.7	0.7	0.8	0.75	0.6
C	0.8	1	0.7	0.7	0.8	0.8	0.9	0.7	0.6
B	0.76	0.7	1	0.9	0.7	0.9	0.6	0.82	0.75
D	0.8	0.7	0.9	1	0.8	0.7	0.9	0.8	0.8
E	0.7	0.8	0.7	0.8	1	0.8	0.75	0.6	0.85
F	0.7	0.8	0.9	0.7	0.8	1	0.9	0.65	0.7
I	0.8	0.9	0.6	0.9	0.75	0.9	1	0.75	0.75
G	0.75	0.7	0.82	0.8	0.6	0.65	0.75	1	0.64
H	0.6	0.6	0.75	0.8	0.85	0.7	0.75	0.64	1

Example of transformed similarity matrix

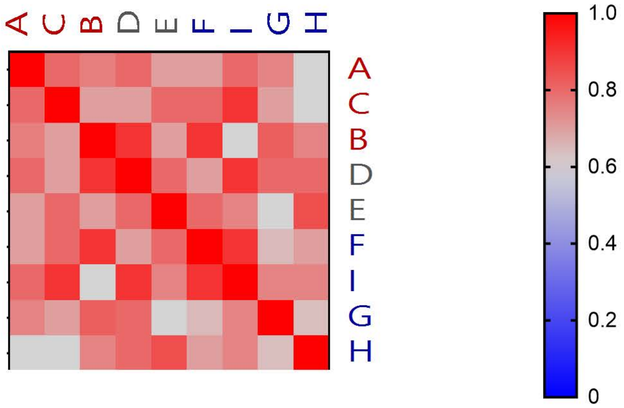

Heatmap of transformed similarity matrix

C

	A	C	B	D	E	F	I	G	H
A	1	1	1	1	1	1	1	1	1
C	1	1	1	1	1	1	1	1	1
B	1	1	1	1	1	1	1	1	1
D	1	1	1	1	1	1	1	1	1
E	1	1	1	1	1	1	1	1	1
F	1	1	1	1	1	1	1	1	1
I	1	1	1	1	1	1	1	1	1
G	1	1	1	1	1	1	1	1	1
H	1	1	1	1	1	1	1	1	1

Ideal transformed similarity matrix

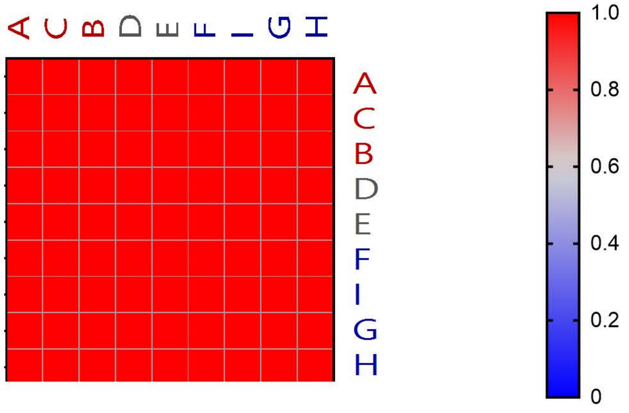

Heatmap of ideal transformed similarity matrix

D Normalized Euclidean distance:

$$NED = \frac{\sqrt{\sum(1-1)^2 + (1-0.8)^2 + (1-0.76)^2 + (1-0.8)^2 + \cdots + (1-1)^2}}{9\ (\#\ samples)}$$

Supplementary Figure 1

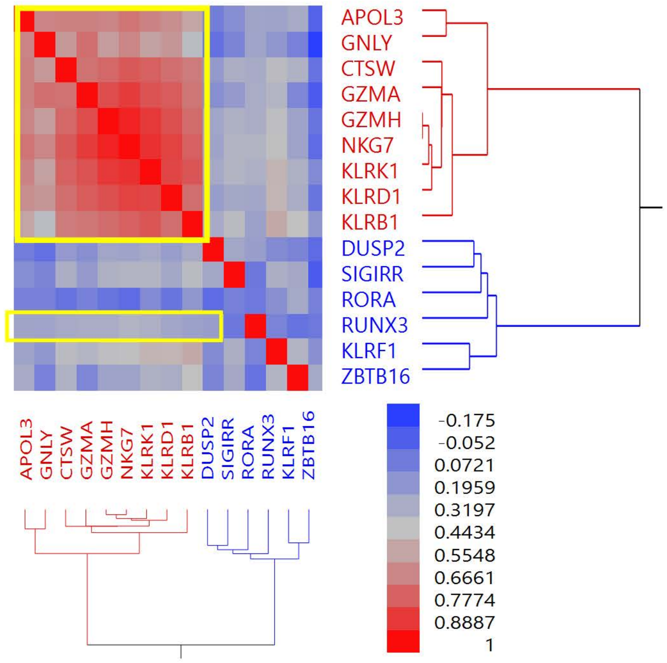

Supplementary Figure 2

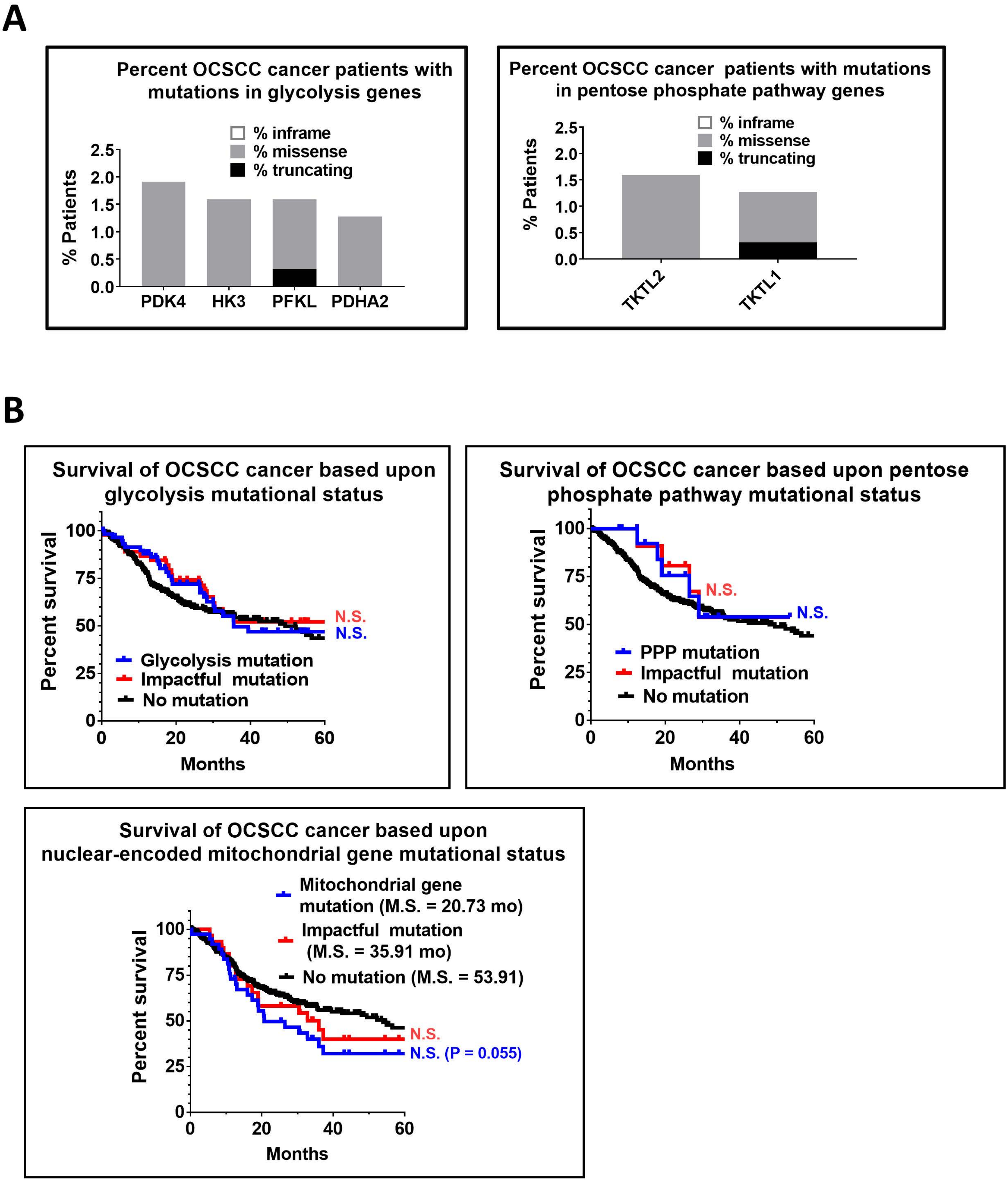

Supplementary Figure 3

A

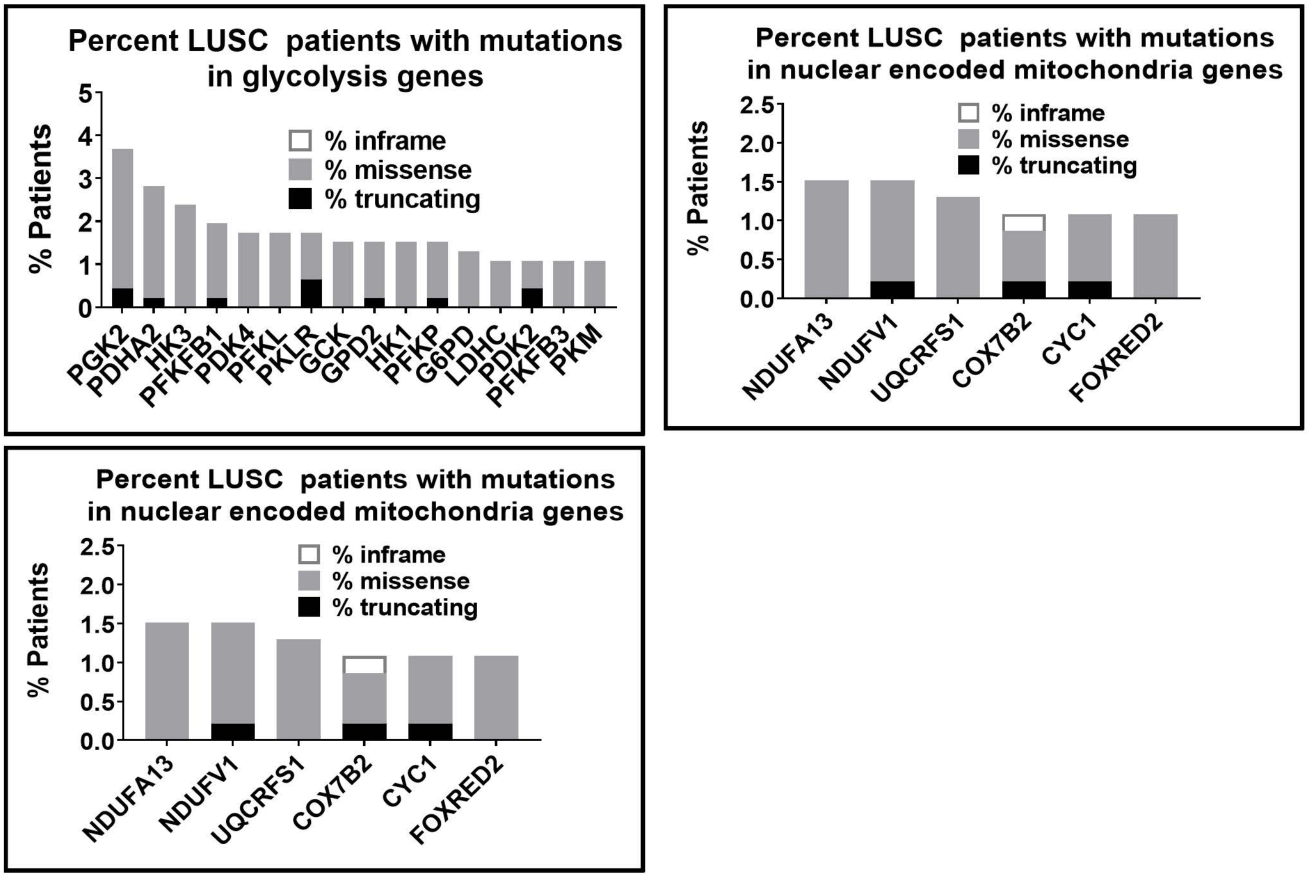

B

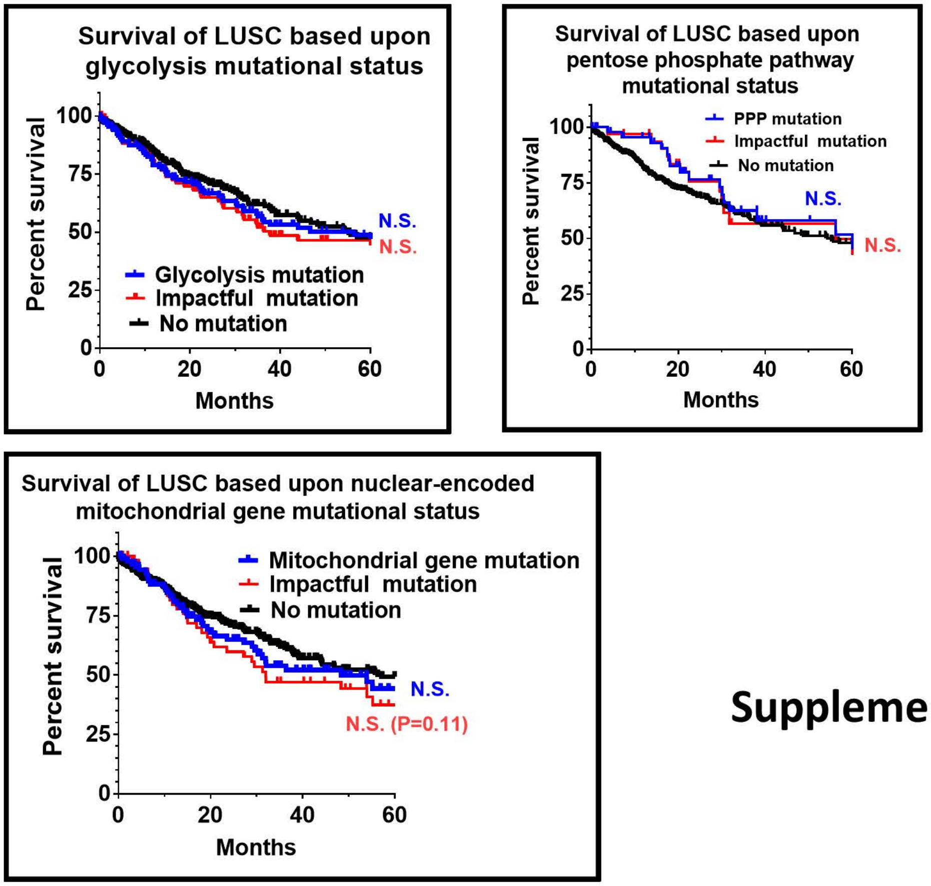

Supplementary Figure 4

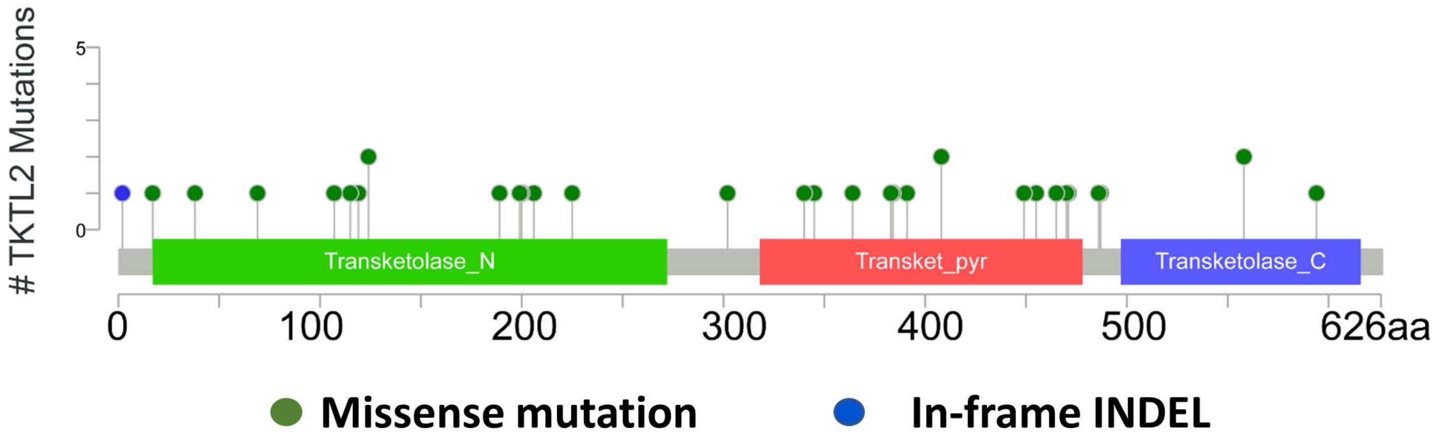

Supplementary Figure 5

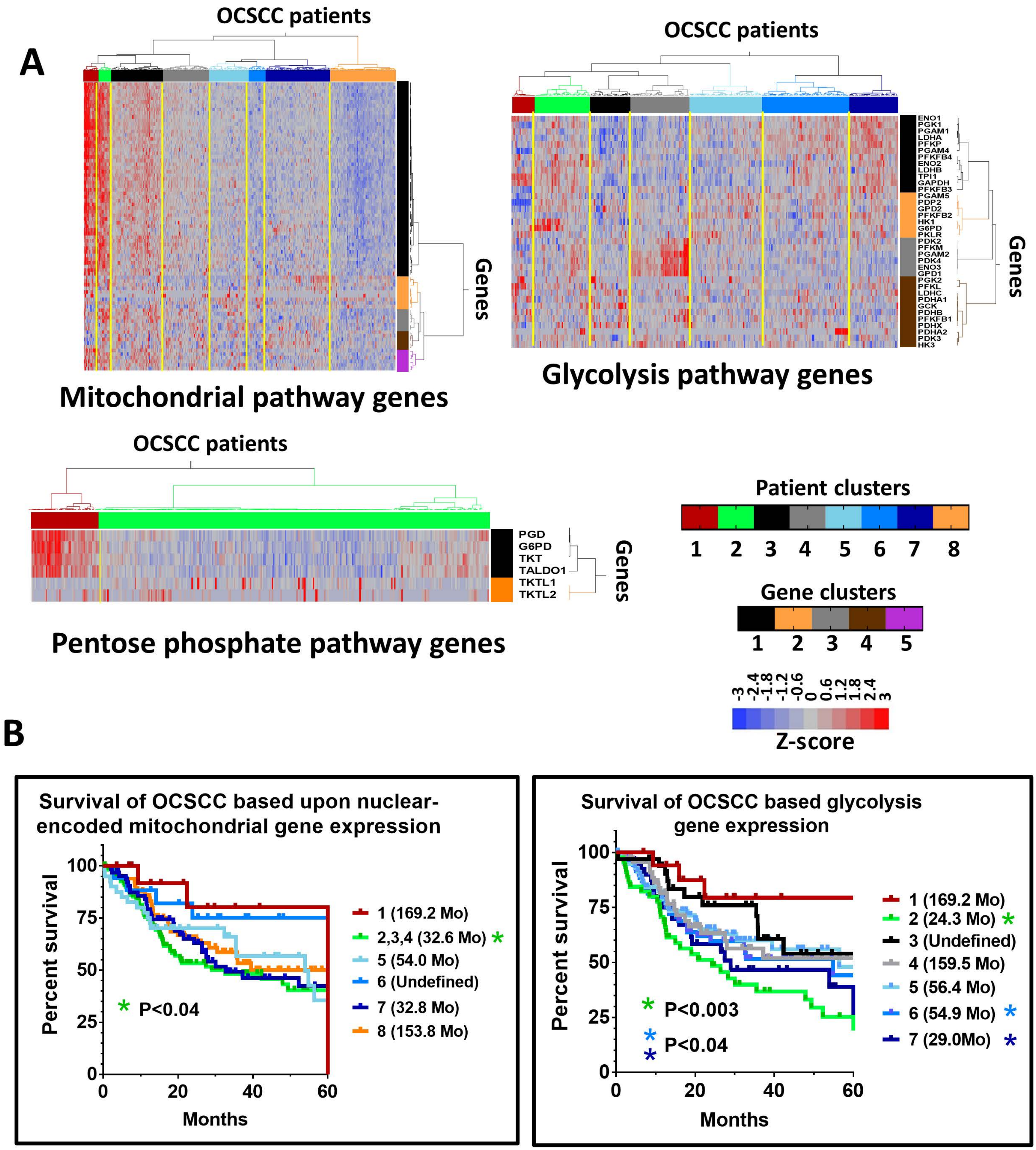

Supplementary Figure 6

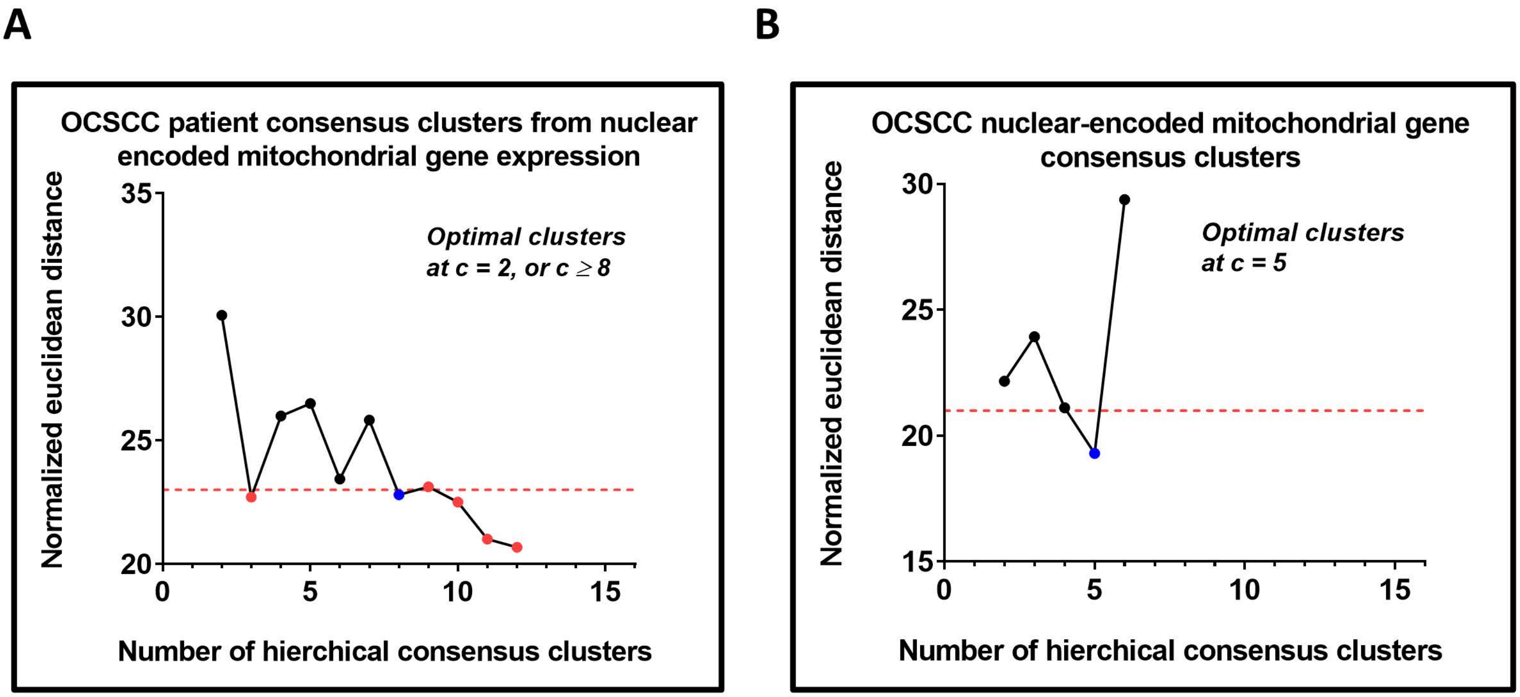

Supplementary Figure 7

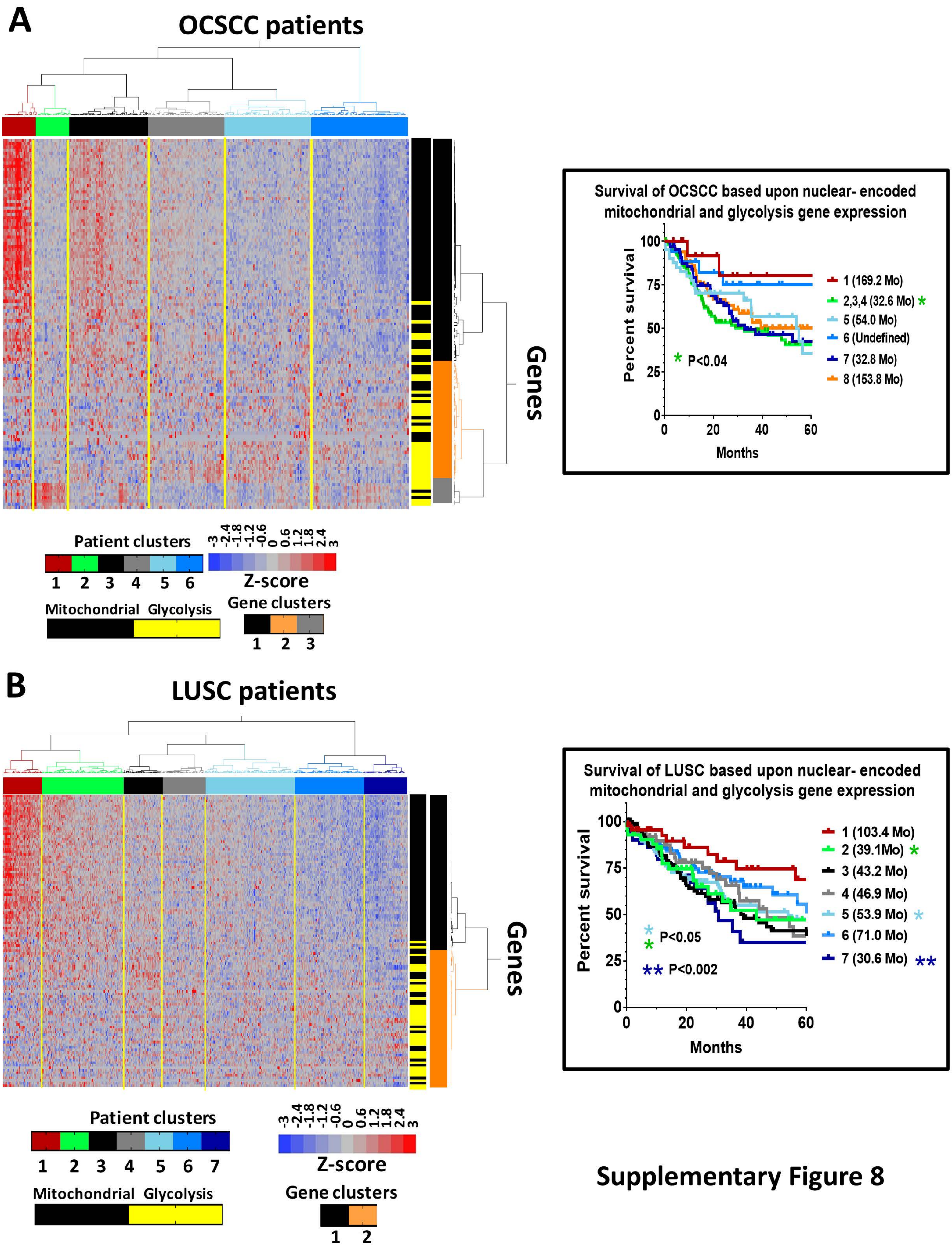

Supplementary Figure 8

Overlap between gene lists

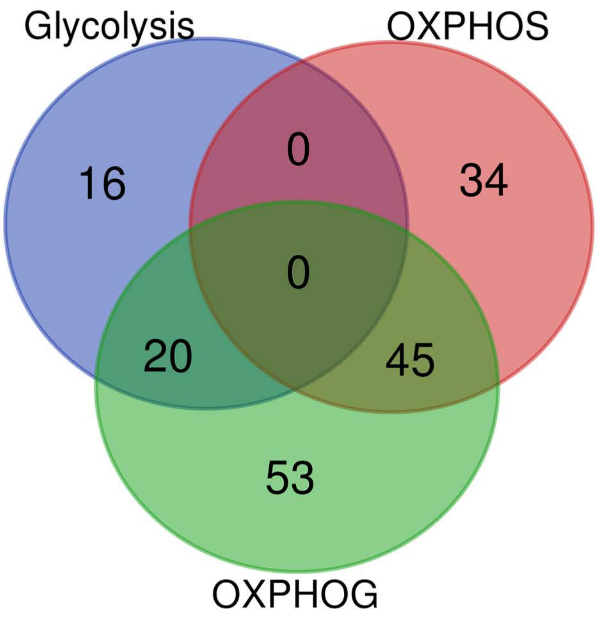

Glycolysis= glycolysis genes (n= 36)
OXPHOS =mitochondrial genes (n= 79)
Gly+ OXPHOS= union of genes (n =115)
OXPHOG= glycolysis and mitochondrial genes from GO networks n= 118)

Supplementary Figure 9

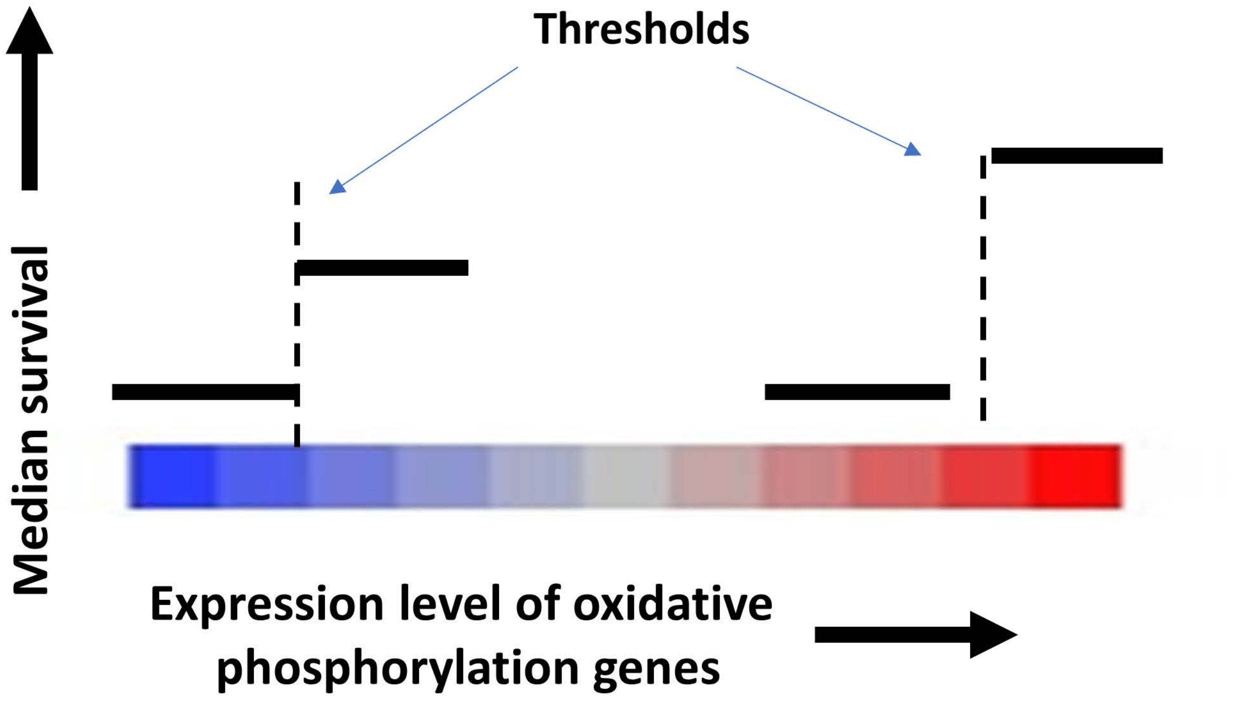

Supplementary Figure 10

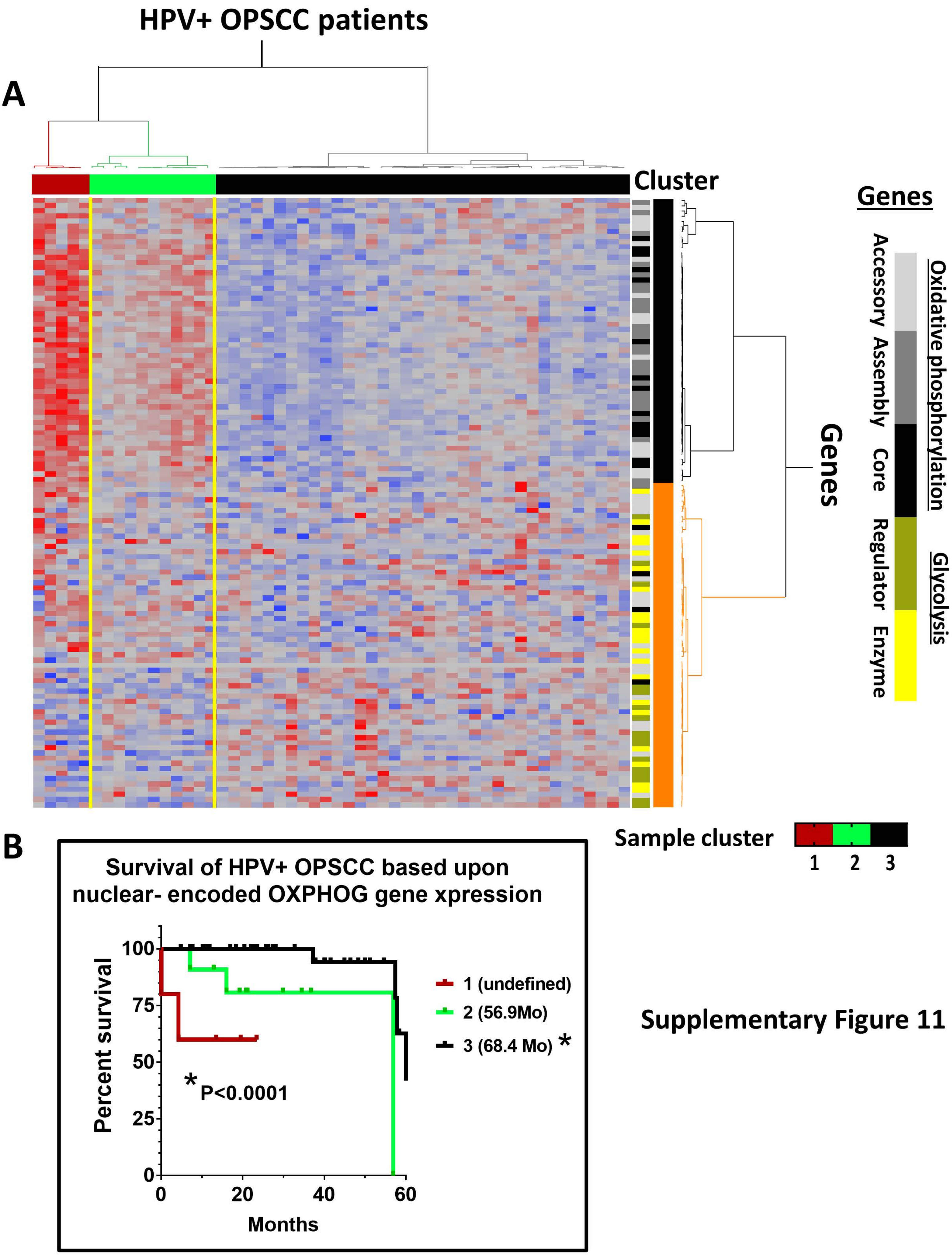

Supplementary Figure 11

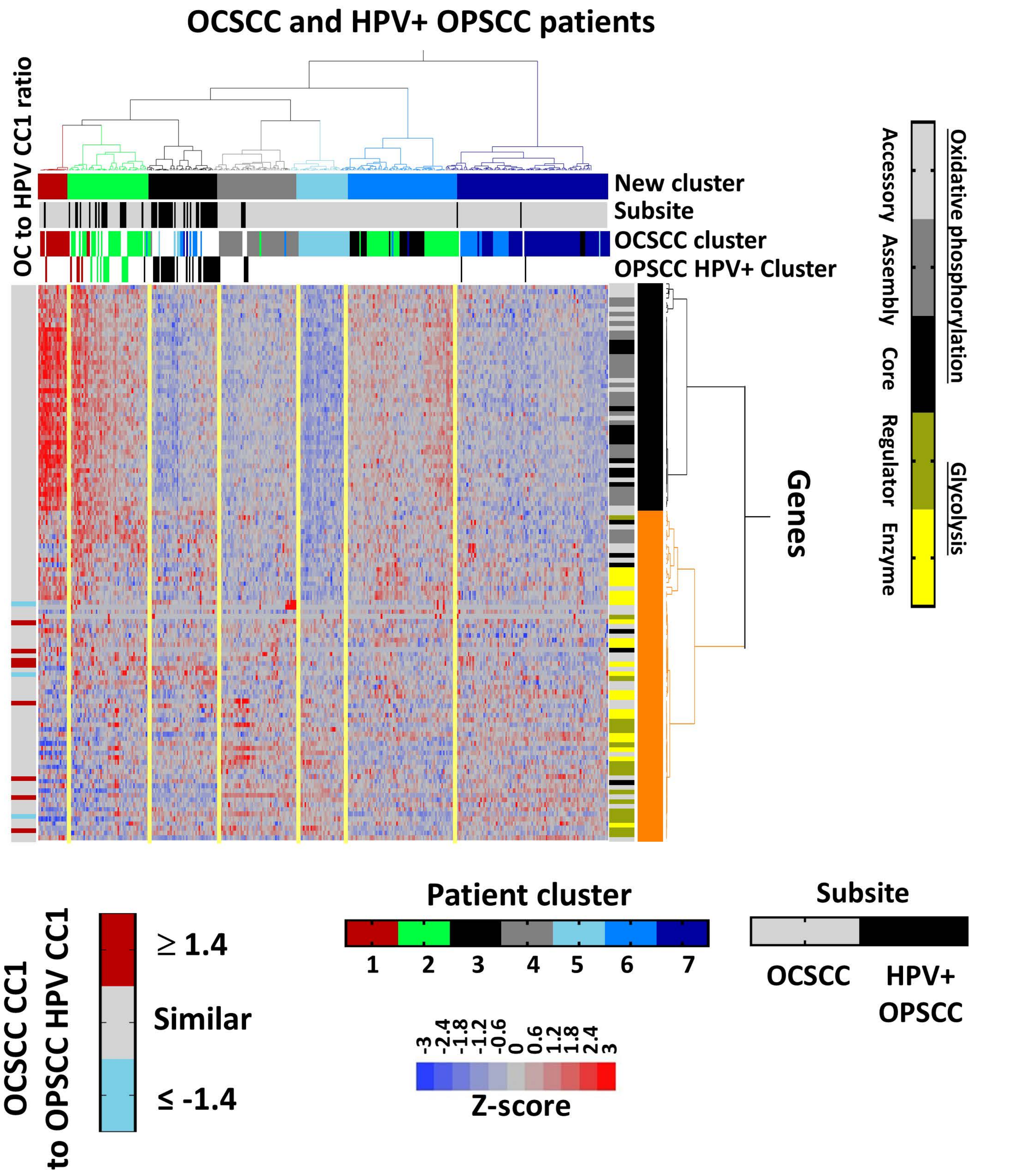

Supplementary Figure 12

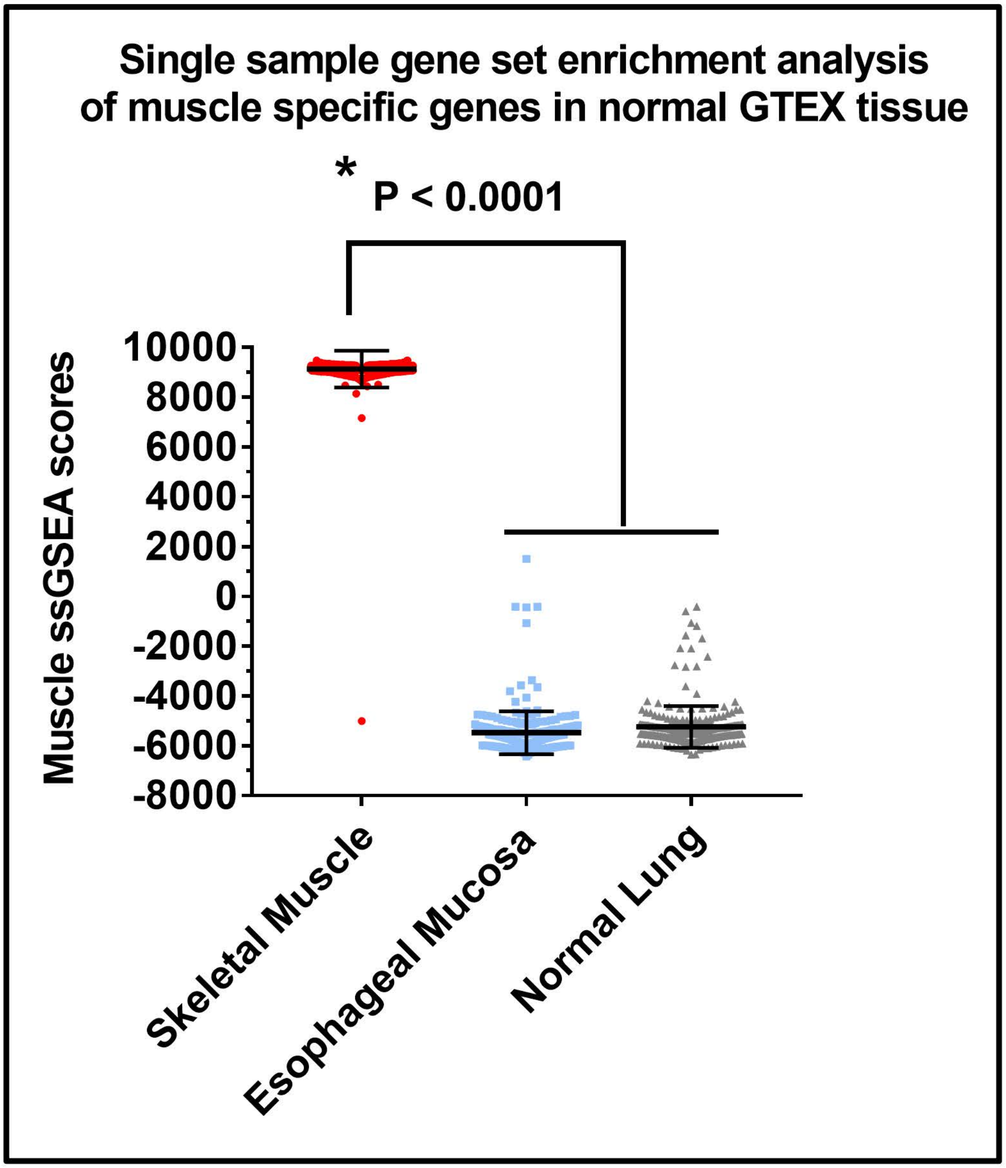

Supplementary Figure 13

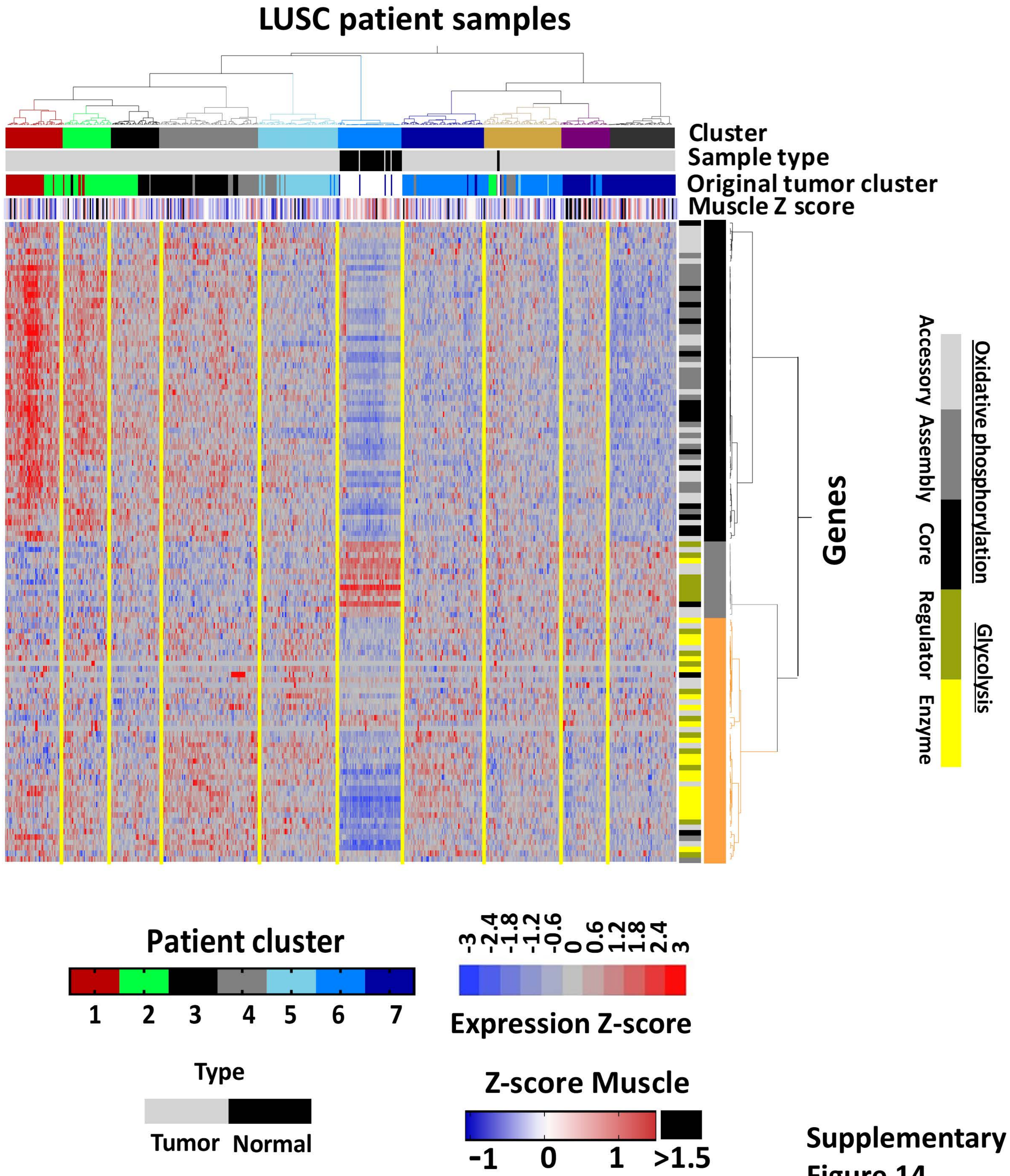

Supplementary Figure 14

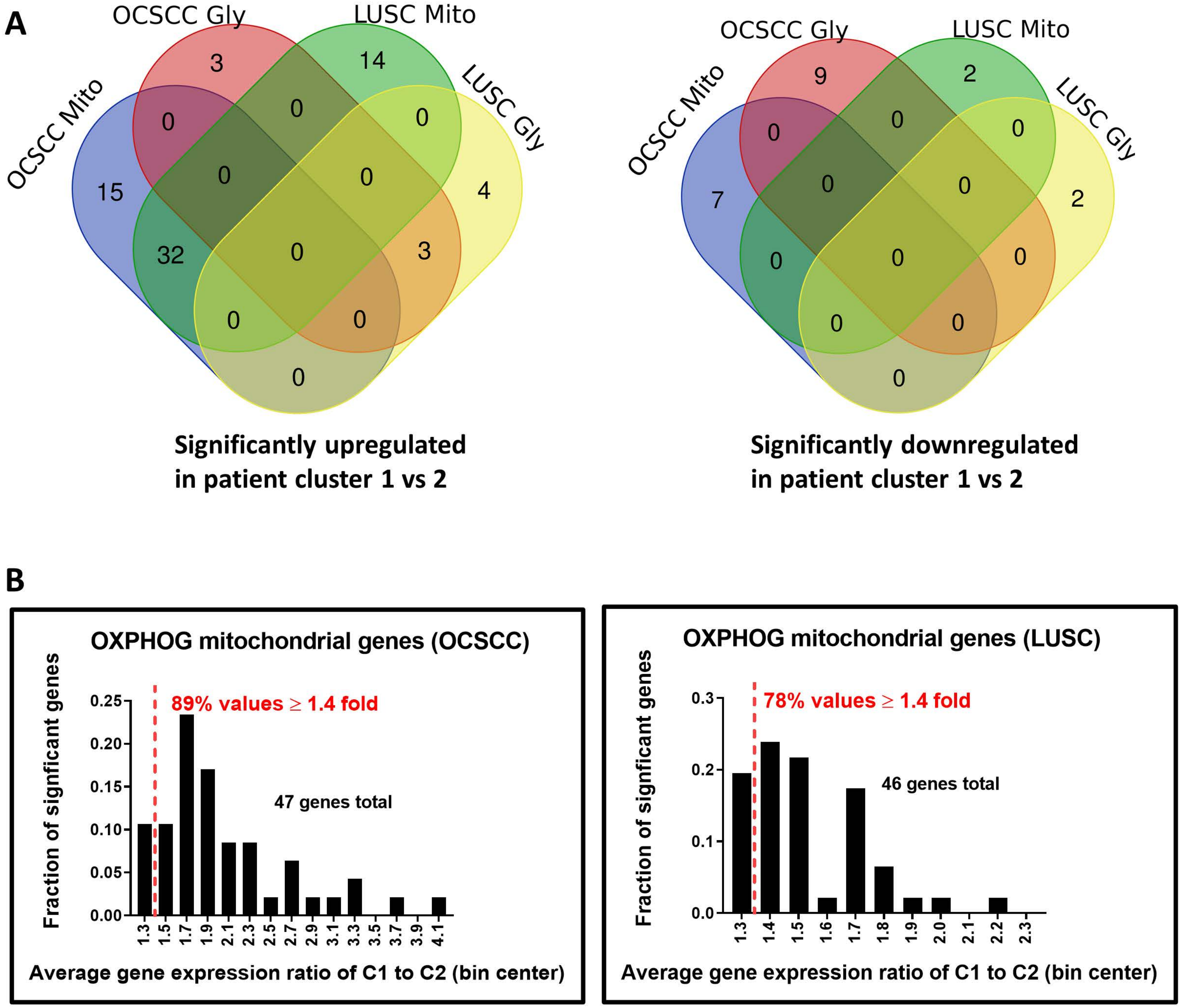

Supplementary Figure 15

A

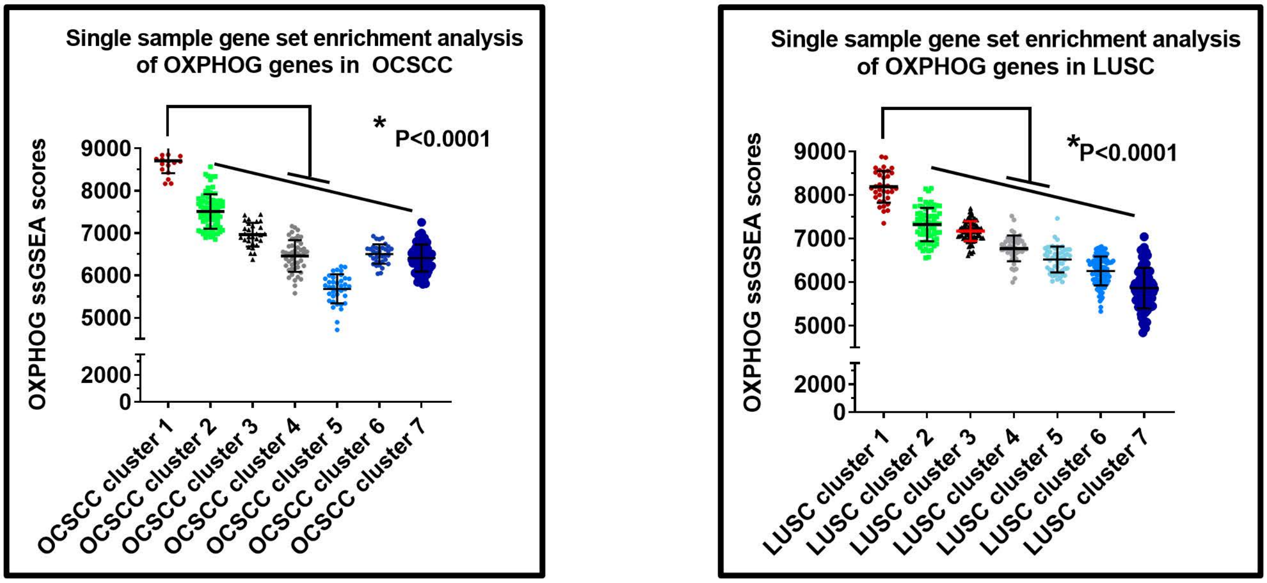

B

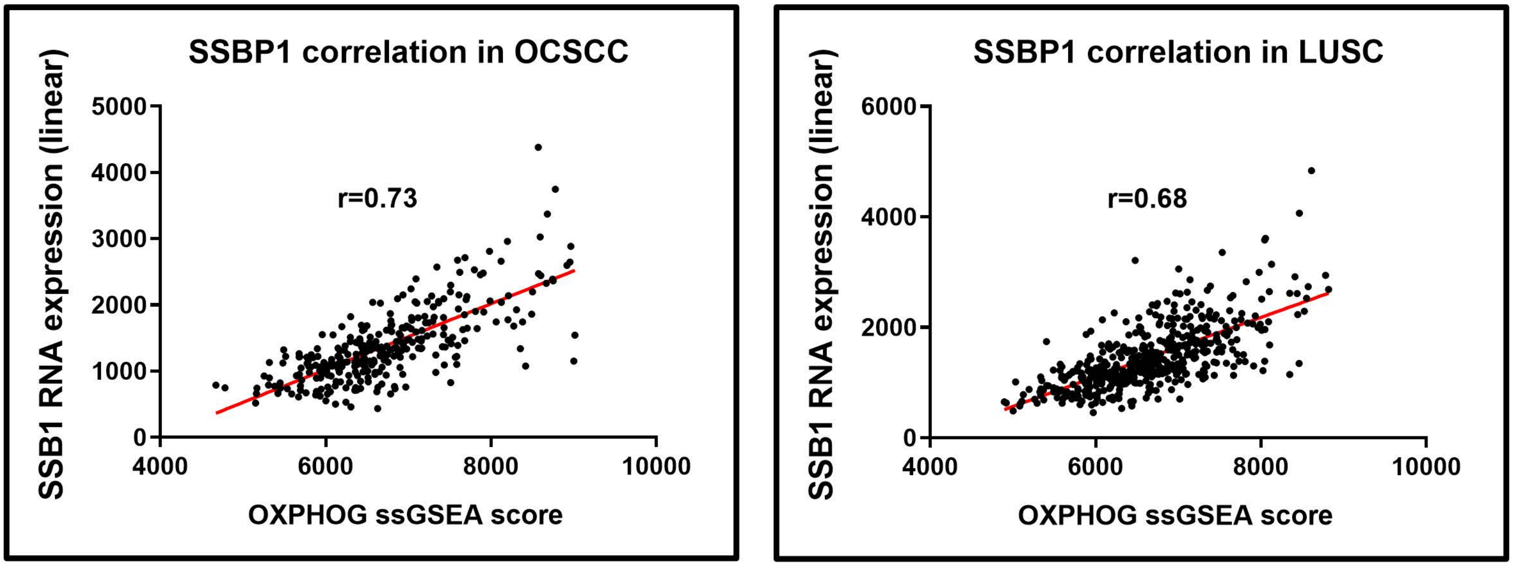

Supplementary Figure 16

A

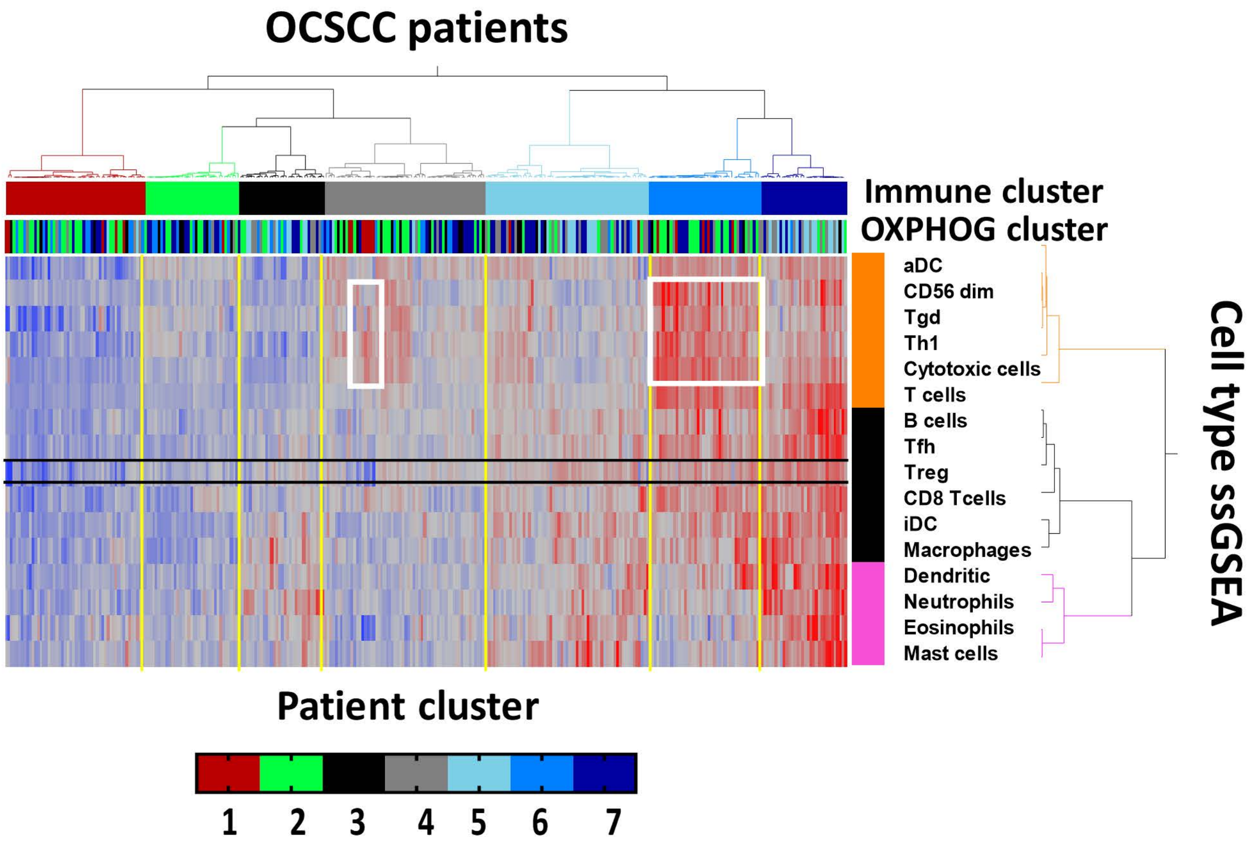

B

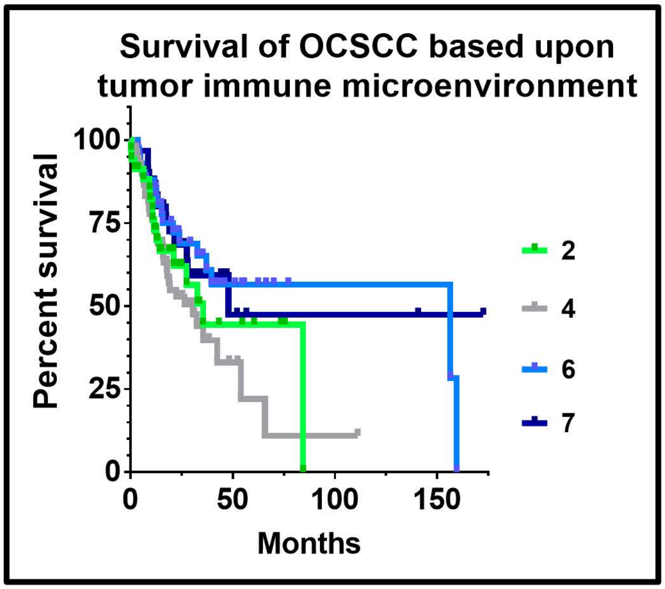

Immune cluster	CC1	CC2	CC3	CC4	CC5	CC6	CC7
Median survival	49.41	35.45	52.27	**30.45**	56.44	**156.37**	47.93
P-value	0.5491	0.181	0.1327	**0.018**	0.7004		0.9806

Supplementary Fig. 17

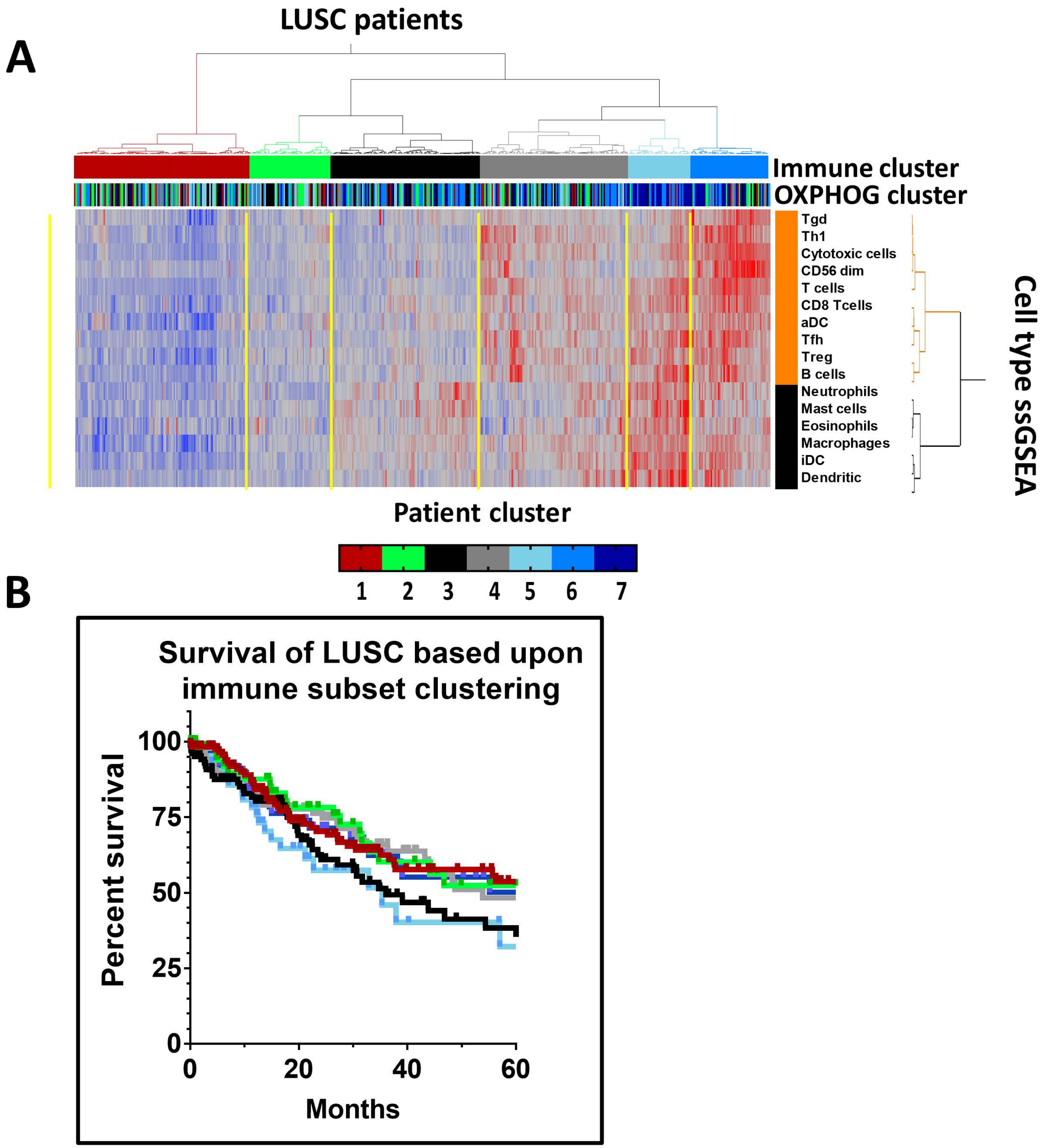

Immune clusters	CC1	CC2	CC3	CC4	CC5	CC6
Median survival	73.06	63.5	36.04	53.88	35.32	64.16
P-value	0.9443	0.9038	0.3007	0.6985	0.2107	

Supplementary Fig. 18

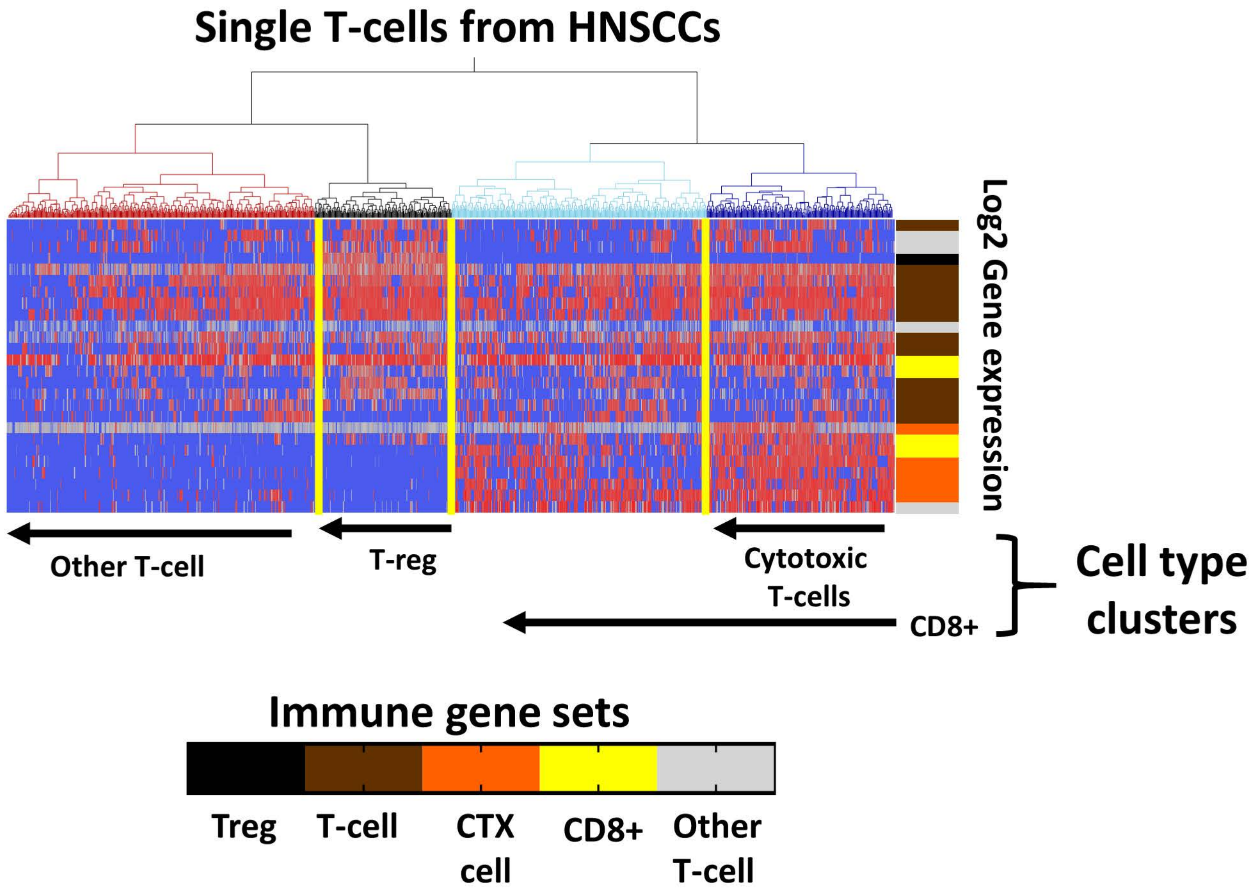

Supplementary Fig. 19
